# Plectin ensures intestinal epithelial integrity and protects colon against colitis

**DOI:** 10.1038/s41385-021-00380-z

**Published:** 2021-03-05

**Authors:** Alzbeta Krausova, Petra Buresova, Lenka Sarnova, Gizem Oyman-Eyrilmez, Jozef Skarda, Pavel Wohl, Lukas Bajer, Eva Sticova, Lenka Bartonova, Jiri Pacha, Gizela Koubkova, Jan Prochazka, Marina Spörrer, Christopher Dürrbeck, Zuzana Stehlikova, Martin Vit, Natalia Ziolkowska, Radislav Sedlacek, Daniel Jirak, Miloslav Kverka, Gerhard Wiche, Ben Fabry, Vladimir Korinek, Martin Gregor

**Affiliations:** 1 Laboratory of Integrative Biology, Institute of Molecular Genetics of the Czech Academy of Sciences, Prague, Czech Republic; 2 Department of Cell Biology, Faculty of Science, Charles University, Prague, Czech Republic; 3 Department of Clinical and Molecular Pathology, Faculty of Medicine and Dentistry, Palacky University and University Hospital in Olomouc, Olomouc, Czech Republic; 4 Institute of Pathology, University Hospital Ostrava, Ostrava, Czech Republic; 5 Department of Gastroenterology and Hepatology, Institute for Clinical and Experimental Medicine, Prague, Czech Republic; 6 Department of Clinical and Transplant Pathology, Institute for Clinical and Experimental Medicine, Prague, Czech Republic; 7 Department of Pathology, Third Faculty of Medicine, Charles University, Prague, Czech Republic; 8 Department of Epithelial Physiology, Institute of Physiology of the Czech Academy of Sciences, Prague, Czech Republic; 9 Czech Centre for Phenogenomics, Institute of Molecular Genetics of the Czech Academy of Sciences, Prague, Czech Republic; 10 Laboratory of Transgenic Models of Diseases, Institute of Molecular Genetics of the Czech Academy of Sciences, Prague, Czech Republic; 11 Department of Physics, University of Erlangen-Nuremberg, Erlangen, Germany; 12 Laboratory of Cellular and Molecular Immunology, Institute of Microbiology of the Czech Academy of Sciences, Prague, Czech Republic; 13 University of Liberec, Faculty of Mechatronics Informatics and Interdisciplinary Studies, Liberec, Czech Republic; 14 Institute of Biophysics and Informatics, First Faculty of Medicine, Charles University, Prague, Czech Republic; 15 Technical University of Liberec, Faculty of Health Studie, Liberec, Czech Republic; 16 Department of Radiodiagnostic and Interventional Radiology, Institute for Clinical and Experimental Medicine, Prague, Czech Republic; 17 Department of Biochemistry and Cell Biology, Max F. Perutz Laboratories, University of Vienna, Vienna, Austria; 18 Laboratory of Cell and Developmental Biology, Institute of Molecular Genetics of the Czech Academy of Sciences, Prague, Czech Republic

## Abstract

Plectin, a highly versatile cytolinker protein, provides tissues with mechanical stability through the integration of intermediate filaments (IFs) with cell junctions. Here, we hypothesize that plectin-controlled cytoarchitecture is a critical determinant of the intestinal barrier function and homeostasis. Mice lacking plectin in an intestinal epithelial cell (IEC; *Ple*^*ΔIEC*^) spontaneously developed colitis characterized by extensive detachment of IECs from the basement membrane (BM), increased intestinal permeability, and inflammatory lesions. Moreover, plectin expression was reduced in the colons of ulcerative colitis (UC) patients and negatively correlated with the severity of colitis. Mechanistically, plectin deficiency in IECs led to aberrant keratin filament (KF) network organization and the formation of dysfunctional hemidesmosomes (HDs) and intercellular junctions. In addition, the hemidesmosomal α6β4 integrin (Itg) receptor showed attenuated association with KFs, and protein profiling revealed prominent downregulation of junctional constituents. Consistent with the effects of plectin loss in the intestinal epithelium, plectin-deficient IECs exhibited remarkably reduced mechanical stability and limited adhesion capacity in vitro. Feeding mice with a low-residue liquid diet that reduced mechanical stress and antibiotic treatment successfully mitigated epithelial damage in the *Ple*^*ΔIEC*^ colon.

## Introduction

The intestinal epithelium is composed of a single layer of tightly linked intestinal epithelial cells (IECs), forming a selective physical barrier that is critical for gut homeostasis. A breach in the intestinal barrier, referred to as “leaky gut”^1^, results in excessive exposure to luminal microbiota and in a concomitant innate immune response. Subsequent dysregulation of the finely-tuned interplay among gut microbiota, IECs, and immune cells accounts for uncontrolled inflammation and pathogenesis of intestinal disorders such as inflammatory bowel disease (IBD) and colorectal cancer (CRC)^2,3^.

The epithelial barrier function is secured by cell junctions that seal intercellular spaces and interlink IECs with the underlying basement membrane (BM) into a structural and functional continuum. Alterations in junctional proteins and BM components may lead to a breakdown of the barrier, and genetic studies identified multiple links between junction/BM-associated genes and the development of IBD^4–6^. While apical tight junctions (TJs) and subjacent adherens junctions (AJs) confer paracellular transport selectivity, desmosomes (Ds) together with BM-linked hemidesmosomes (HDs) provide the intestinal epithelium with resilience to mechanical stress generated by intestinal peristalsis^7^. It is noteworthy that recently reported mouse models demonstrate the protective role of Ds and HDs in the context of both intestinal inflammation^8,9^ and colitis-associated CRC^8^. Accumulating evidence suggests that fundamental features of Ds and HDs (such as stability, dynamics, and mechanotransduction capacity) heavily rely on their interconnection with keratin filament (KF) networks^10,11^. This places plakins^12^, a family of cytolinker proteins mediating physical linkage between KFs and cell junctions, at the very center of the processes controlling epithelial homeostasis.

Plectin, a highly versatile member of the plakin protein family, crosslinks intermediate filaments (IFs) of different types and anchors them at cellular junctions, including HDs and Ds of epithelial cells^13^. Multiple mutations in the *plectin* gene have been identified in epidermolysis bullosa (EB)^14^, a disorder characterized by excessive blister formation in skin^15,16^ with reported cases of concurrent IBD^17,18^. Previous studies have shown that plectin ablation disrupts highly organized epithelial KF networks and alters the structure and functionality of cell junctions^19–22^. For example, tissue-specific deletion of *plectin* in the mouse biliary epithelium has adverse effects on the formation of TJs, AJs, and Ds, with deleterious consequences for epithelial stability under cholestasis^22^. Likewise, analysis of knock-in mice recapitulating dominant EB simplex suggests that HD stability in basal keratinocytes depends on plectin-mediated recruitment of KFs^20^. Mechanistically, dysfunctional HDs account for epithelial fragility and lesional defects^23^ which resemble those seen in patients with IBD^24^. Although these observations suggest a linkage between plectin dysfunction and intestinal pathologies, plectin’s role in the intestinal epithelium remains unaddressed.

In this study, we found that plectin expression was reduced in patients with active ulcerative colitis (UC) and that plectin expression levels negatively correlated with the severity of colitis. To study the underlying molecular mechanisms, we generated two new mouse lines: one constitutive (*Ple*^*ΔIEC*^) and the other with tamoxifen (TMX)-inducible (*Ple*^*ΔIEC-ERT2*^) plectin ablation in IECs. The phenotypic characterization of these mice demonstrated that loss of plectin leads to spontaneous development of a colitic phenotype characterized by extensive detachment of IECs from the BM, increased intestinal permeability, and formation of inflammatory lesions. These results demonstrate the absolute indispensability of plectin for the maintenance of intestinal epithelium integrity, and moreover that both mouse lines provide a useful model system for investigating disease etiology and testing palliative therapies.

## Results

### Suppression of plectin in human patients with UC

**Fig. 1:**
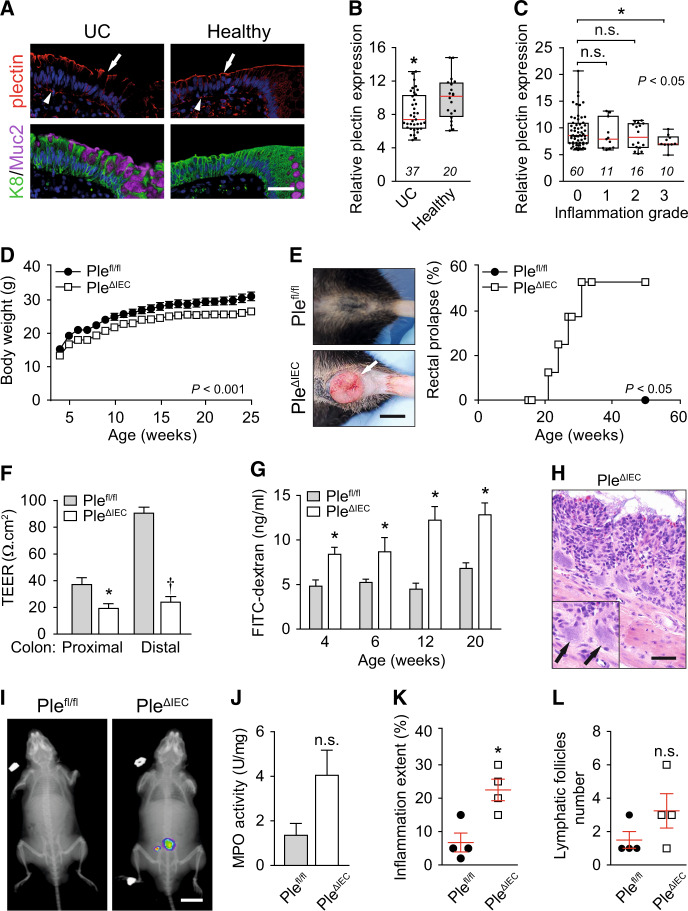
Loss of plectin is associated with UC in human patients and leads to intestinal epithelial barrier dysfunction with concomitant inflammation in mouse. **A** Paraffin-embedded colon sections from UC patients (UC) and healthy controls (healthy) were immunolabeled with antibodies to plectin (red), keratin 8 (K8; green), and mucin 2 (Muc2; magenta). Nuclei were stained with Hoechst (blue). Arrows, apical IEC membrane; arrowheads, basal IEC membrane. Scale bar, 40 μm. **B** Relative *plectin* mRNA levels in rectum biopsies collected from healthy controls and patients with active UC. Scattered boxplots show individual data points, median, 25th, and 75th percentile with whiskers reaching the last data point. The numbers of included participants per cohort are indicated in the graph. **C** Relative *plectin* mRNA expression in rectum biopsies collected from UC patients clustered based on inflammation scored in H&E-stained rectum sections. Scattered boxplots show individual data points, median, 25th, and 75th percentile with whiskers reaching the last data point. The numbers of included participants per cohort are indicated in the graph. **D** Bodyweight of *Ple*^*fl/fl*^ and *Ple*^*ΔIEC*^ mice was monitored for 25 weeks, *n* = 7. **E** Representative images of the rectum of 30-week-old *Ple*^*fl/fl*^ and *Ple*^*ΔIEC*^ mice. Kaplan–Meier graph shows age-related rectal prolapse incidence. **F** Intestinal transepithelial electrical resistance (TEER) measured ex vivo in both proximal and distal colons of 12-week-old *Ple*^*fl/fl*^ and *Ple*^*ΔIEC*^ mice, *n* = 4. **G** In vivo permeability of mucosa of *Ple*^*fl/fl*^ and *Ple*^*ΔIEC*^ mice (at the age indicated) measured by monitoring 40-kDa FITC-dextran levels in plasma 4 h after orogastric gavage, *n* = 3–7. **H** Representative image of *Ple*^*ΔIEC*^ colon section from 30-week-old *Ple*^*ΔIEC*^ mouse stained with H&E. Arrows, bacterial patches in the mucosa. Scale bar, 50 μm. **I** In vivo chemiluminescence images of 12-week-old *Ple*^*ΔIEC*^ and *Ple*^*fl/fl*^ mice injected with myeloperoxidase (MPO) inflammation probe. **J** MPO activity (a marker of neutrophil infiltration) measured in colon lysates from 12-week-old *Ple*^*ΔIEC*^ and *Ple*^*fl/fl*^ mice, *n* = 3. **K**, **L** Inflammation extent (percentage) (**K**) and the number of lymphatic follicles (**L**) assessed from H&E-stained sections of 12-week-old *Ple*^*fl/fl*^ and *Ple*^*ΔIEC*^ colons, *n* = 4. Data are presented as mean ± SEM, n.s. not significant, *P < 0.05, **P < 0.01, ^†^*P* < 0.001.

To examine the role of plectin in the pathogenesis of UC, we screened for potential alterations of plectin expression in a cohort of ~100 UC patients. The analysis of immunolabeled biopsy samples taken from patients and healthy controls revealed discontinuous and rather patchy plectin staining patterns in UC biopsies. The gaps in the plectin staining pattern coincided with goblet cell openings heavily loaded with mucus. In healthy controls, plectin decorated both apical and basal membranes of IECs evenly (Figs. 1A and S1A), resembling plectin localization in mouse intestinal sections (Fig. S1B and published previously^25^). In addition, mRNA profiling showed significantly reduced expression levels of plectin in biopsies from patients with active UC (Fig. 1B). Histological analysis revealed that low mRNA levels of plectin were associated with higher inflammation (Fig. 1C) and higher C reactive protein levels in serum (not shown).

### IEC-specific plectin-deficient mice develop a colitic phenotype due to intestinal barrier dysfunction

To explore the role of plectin in the intestinal epithelium in greater detail, we generated IEC-specific *plectin* knockout (*Ple*^*ΔIEC*^) mice. Successful ablation of plectin in IECs was confirmed by immunofluorescence microscopy (Fig. S1C). The newly generated *Ple*^*ΔIEC*^ mice had a considerably lower bodyweight (Figs. 1D and S1D), suffered from persistent diarrhea with occasional rectal bleeding (Fig. S1E, and not shown), and frequently developed rectal prolapse (Fig. 1E). As the onset and progression of UC correlate with defects in the intestinal barrier function^26,27^, we assessed barrier integrity either by ex vivo measurements of intestinal transepithelial electrical resistance (TEER) or by in vivo orogastric gavage of FITC-dextran. We observed significantly lower TEER in the proximal and distal colon regions of 12-week-old *Ple*^*ΔIEC*^ compared to *Ple*^*fl/fl*^ mice. Moreover, TEER values in *Ple*^*fl/fl*^ mice were three-times higher in their distal parts than in their proximal parts; by contrast, TEER values in *Ple*^*ΔIEC*^ mice were equally low in distal and proximal colon segments (Fig. 1F). Compared to *Ple*^*fl/fl*^ mice, *Ple*^*ΔIEC*^ mice consistently displayed a higher penetration rate of FITC-dextran into blood already at 4 weeks, and this difference became even more apparent in older animals (Fig. 1G).

In addition, a histological inspection of hematoxylin-eosin (H&E)-stained colon sections revealed extensive translocation of luminal bacteria into *Ple*^*ΔIEC*^ mucosa in 30-week-old mice (Fig. 1H). Given the hampered barrier function in the *Ple*^*ΔIEC*^ intestine, we screened *Ple*^*fl/fl*^ and *Ple*^*ΔIEC*^ mice for signs of inflammation. Chemiluminescence-based whole body imaging^28^ showed positive abdominal areas in *Ple*^*ΔIEC*^ mice (Fig. 1I), which correlated strongly with significantly higher myeloperoxidase activity (MPO; Fig. 1J). Moreover, mild inflammation of the *Ple*^*ΔIEC*^ colon was confirmed by increased immune cell infiltration, extent (or intensity) of acute/chronic inflammation, and lymphatic follicle size (Figs. 1K, L and S2A), and a higher percentage of edema and ulceration indicated higher epithelial damage (Fig. S2B). Together, these results suggest that plectin is critical for the maintenance of the intestinal barrier and thus could be directly linked to the onset and progression of UC.

### Loss of plectin leads to hyperproliferation and aberrant differentiation of IECs

**Fig. 2:**
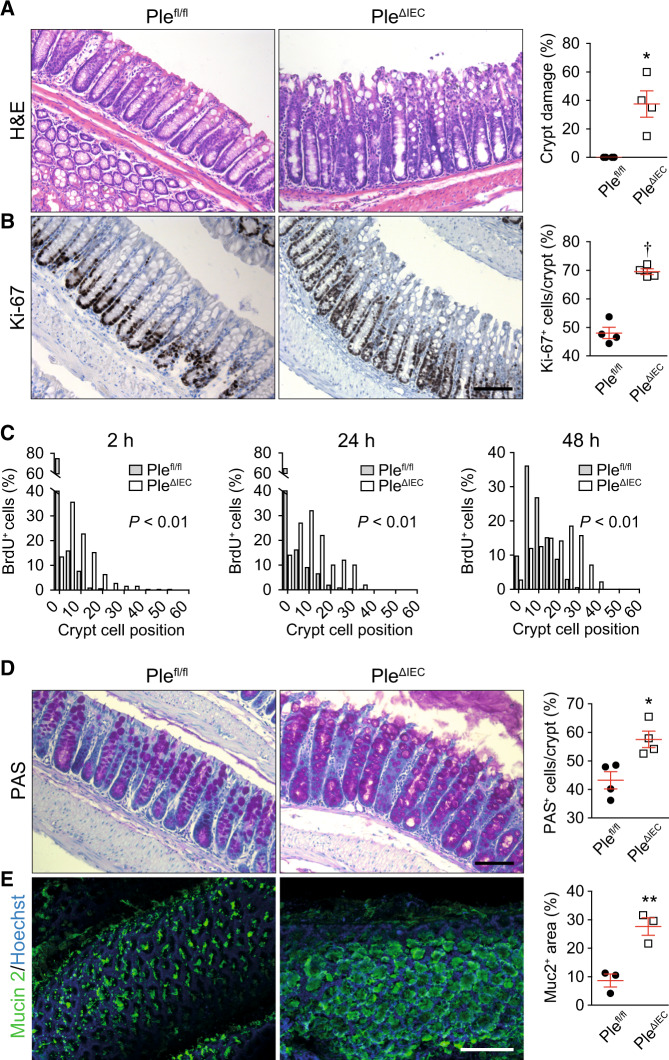
Plectin-deficient IECs exhibit aberrant proliferation and differentiation, resulting in altered crypt organization. **A**, **B** Representative images of H&E staining (**A**) and Ki-67 immunohistochemistry (proliferating cells) (**B**) of *Ple*^*fl/fl*^ and *Ple*^*ΔIEC*^ paraffin-embedded colon sections. Scale bar, 100 μm. Graphs show quantification of colonic crypt damage given as a percentage of crypts with >5% of IECs detached from BM (**A**) and percentage of the Ki-67-positive (Ki-67^+^) IECs per crypt (**B**), *n* = 3–4. **C** Histograms showing the percentage of BrdU-positive (BrdU^+^) cells in given positions of *Ple*^*fl/fl*^ and *Ple*^*ΔIEC*^ colonic crypts at 2, 24, and 48 h after BrdU pulse. Cells were numbered sequentially from crypt base to lumen, with cell position 0 assigned to the first cell at the base of each crypt. At least nine crypts per mouse were analyzed from three mice per time point and genotype. **D**, **E** Representative images of PAS staining (goblet cells) (**D**) and mucin-2 (Muc2) immunofluorescence in mucus layer (**E**) of *Ple*^*fl/fl*^ and *Ple*^*ΔIEC*^ distal colon sections (**D**) and colon whole mounts (**E**). Scale bars, 100 μm (**D**), and 200 μm (**E**). Graphs show quantification of percentage of PAS-positive (PAS^+^) IECs per crypt (**D**) and percentage of mucin-2-positive (Muc2^+^) area per whole mount area examined (**E**), *n* = 3–4. Data are presented as mean ± SEM, *P < 0.05, **P < 0.01, ^†^*P* < 0.001.

Further histological inspection of H&E-stained colonic sections revealed thickening of the colonic mucosa and significant crypt damage with excessive sloughing of IECs detached from the subjacent BM in plectin-deficient specimens (Fig. 2A). In addition, the colon of *Ple*^*ΔIEC*^ mice showed a higher rate of proliferation as determined from Ki-67-stained sections (Fig. 2B). Consistently, an increase in the number of proliferating transit-amplifying IECs in the crypts of *Ple*^*ΔIEC*^ animals was evident from BrdU incorporation assessed 2, 24, and 48 h after a BrdU pulse (Fig. 2C). Interestingly, TUNEL staining indicated a minimal degree of spontaneous apoptosis in both *Ple*^*fl/fl*^ and *Ple*^*ΔIEC*^ mice (Fig. S3A). In parallel with the prominent hyperplasia, the *Ple*^*ΔIEC*^ colon contained a higher proportion of PAS-positive goblet cells (Fig. 2D), corresponding to a higher mucus discharge (Fig. 2E). Immunolabeled *Ple*^*ΔIEC*^ colonic sections also showed a lower percentage of chromogranin A (ChgA)-positive enteroendocrine cells (Fig. S3B) and an extended keratin 20 (K20)-positive zone (Fig. S3C). Similar, albeit less pronounced, trends were observed in the *Ple*^*ΔIEC*^ small intestine (Fig. S4). Plectin deficiency thus results in hyperproliferation and aberrant differentiation of IECs, affecting the spatiotemporal organization of the intestinal epithelium.

### Plectin-deficient IECs form aberrant cell junctions and disordered KF networks

**Fig. 3:**
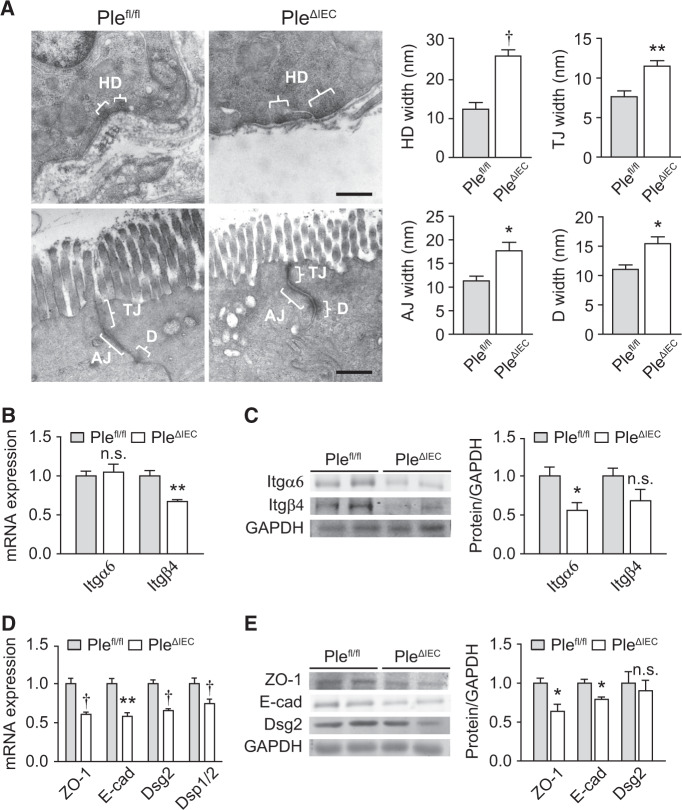
Formation of aberrant cell junctions in *Ple*^*ΔIEC*^ IECs. **A** Representative TEM micrographs of *Ple*^*fl/fl*^ and *Ple*^*ΔIEC*^ IEC junctional complexes. Braces (white) indicate hemidesmosomes (HD), tight junctions (TJ), adherens junctions (AJ), and desmosomes (Ds). Scale bar, 500 nm. Graphs show quantitative analyses of junctional complex widths (measured as the distance from IEC to BM (HD) or distance from IEC to IEC membrane (TJ, AJ, and Ds)). Five to fifteen junctions were measured (two mice per genotype). **B** Relative mRNA levels of integrin (Itg) α6 and β4 in scraped mucosa from *Ple*^*fl/fl*^ and *Ple*^*ΔIEC*^ distal colons, *n* = 4–5. **C** Quantification of Itgβ4 and Itgα6 in scraped distal colon mucosa from *Ple*^*fl/fl*^ and *Ple*^*ΔIEC*^ mice by immunoblotting. GAPDH, loading control. The graph shows relative band intensities normalized to average *Ple*^*fl/fl*^ values, *n* = 3. **D** Relative mRNA levels of ZO-1, E-cadherin (E-cad), desmoglein 2 (Dsg2), and desmoplakin 1/2 (Dsp1/2) in *Ple*^*fl/fl*^ and *Ple*^*ΔIEC*^ distal colons, *n* = 5. **E** Quantification of ZO-1, E-cad, and Dsg2 in *Ple*^*fl/fl*^ and *Ple*^*ΔIEC*^ colon mucosa by immunoblotting. GAPDH, loading control. The graph shows relative band intensities normalized to average *Ple*^*fl/fl*^ values, *n* = 3. Data are presented as mean ± SEM, n.s. not significant, *P < 0.05, **P < 0.01, ^†^*P* < 0.001.

As the structural and functional integrity of epithelia is secured by cell junctions^2,29^, we compared the morphology of cell-ECM (HDs) and cell–cell (TJs, AJs, and Ds) adhesions formed by *Ple*^*fl/fl*^ and *Ple*^*ΔIEC*^ IECs, using transmission electron microscopy (TEM). A quantitative analysis of the HD size revealed an extended cross-sectional length of seemingly less electrodense HD plaques in the *Ple*^*ΔIEC*^ colon; furthermore, the space between HDs and the BM was significantly dilated (Fig. 3A). Similar to HDs, we also found significantly dilated intercellular spaces of TJs, AJs, and Ds between adjacent *Ple*^*ΔIEC*^ IECs (Fig. 3A). These morphological alterations coincided with generally lower expression levels of the hemidesmosomal constituents Itgα6 and Itgβ4 and the following cell–cell junctional markers: zonula occludens 1 (ZO-1; TJs), E-cadherin (E-cad; AJs), desmoglein 2 (Dsg2; Ds), and desmoplakin 1/2 (Dsp1/2; also Ds) at both mRNA and protein levels (Fig. 3B–E). These results clearly show that plectin deficiency leads to the formation of aberrant intestinal junctional complexes, which likely accounts for breached epithelial barrier integrity.

**Fig. 4:**
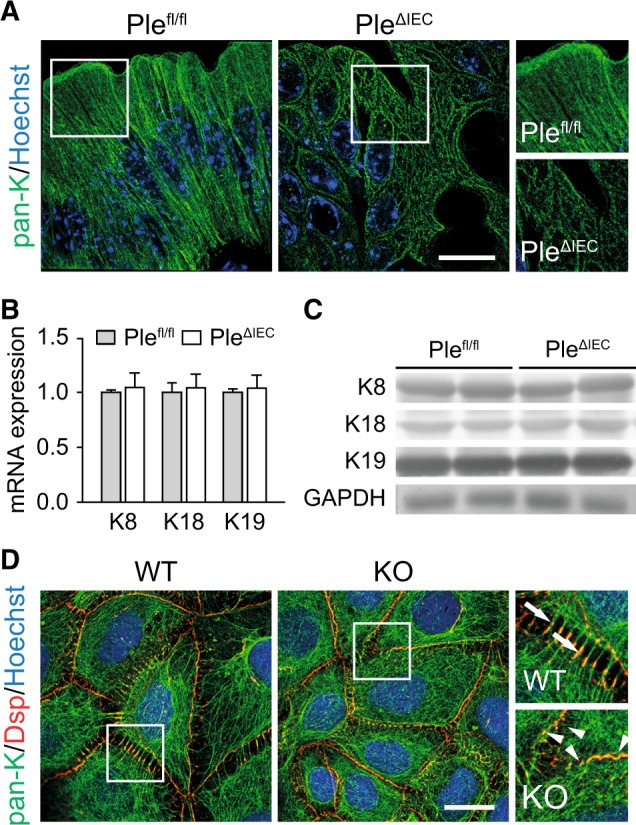
Plectin organizes KFs in IECs. **A** Representative super-resolution STED images of *Ple*^*fl/fl*^ and *Ple*^*ΔIEC*^ distal colon sections immunolabeled for pan-keratin (pan-K; green) with nuclei stained with Hoechst (blue). Scale bar, 10 μm. Boxed areas show ×1.3 images. **B**, **C** Relative mRNA (**B**) and protein (**C**) levels of K8, 18, and 19 in *Ple*^*fl/fl*^ and *Ple*^*ΔIEC*^ distal colon, *n* = 3–5. Data are presented as mean ± SEM, *P* > 0.05 by unpaired Student *t* test. **D** Representative immunofluorescence images of WT and KO Caco-2 cell monolayer cultures immunolabeled for pan-K (green) and desmoplakin (Dsp; red). Nuclei were stained with Hoechst (blue). Arrows, straight K8 filaments anchored to Dsp-positive desmosomes; arrowheads, tangled K8 filaments. Scale bar, 20 μm. Boxed areas show ×2.5 images.

In previous studies, we showed that plectin controls cell junctions through anchorage of IF networks^20,22,30^. Therefore, we next compared the appearance of KFs in *Ple*^*fl/fl*^ and *Ple*^*ΔIEC*^ colon sections using immunofluorescence microscopy. Although the general appearance of K8 and K19 networks did not significantly differ in the two cell types (Fig. S5), super-resolution microscopy of pan-K-labeled sections revealed less pronounced apical staining of *Ple*^*ΔIEC*^ IECs (Fig. 4A). Moreover, in *Ple*^*ΔIEC*^ IECs, pan-K positive filaments formed less-ordered and rather coarse meshworks, while *Ple*^*fl/fl*^ IECs displayed typical staining patterns with filaments regularly aligned along the apicobasal axis (Fig. 4A). The changes in KF network organization were not caused by altered keratin (K8, K18, and K19) expression, as no differences were found at either the mRNA or the protein level (Fig. 4B, C). No apparent abnormalities were seen in actin filament and microtubule organization (Figs. S5 and S6A).

Aberrant KF cytoarchitecture was also clearly discernible in pan-K immunolabeled monolayers of plectin-deficient (KO) human IECs (Caco-2). To mimick the in vivo situation, mature differentiated Caco-2 cells 16 days after the confluency were used. In wild-type (WT) cells, the KF network was densely packed around the cell center, from which individual KFs extended towards the cell periphery delineated by clearly defined desmoplakin-positive Ds (Fig. 4D). In contrast, KO cells showed tangled KFs, which were evenly distributed throughout the cytoplasm and seemingly overlapped with rather continuous desmoplakin-positive structures at the cell–cell borders (Fig. 4D). Similar to *Ple*^*ΔIEC*^ IECs, actin organization in KO cells appeared inconspicuous (Fig. S6B). Collectively, these findings indicate that plectin ablation in IECs results in altered keratin network organization and aberrant KF anchorage to desmosomal junctions.

### Plectin preserves intestinal epithelial integrity through HD stabilization

**Fig. 5:**
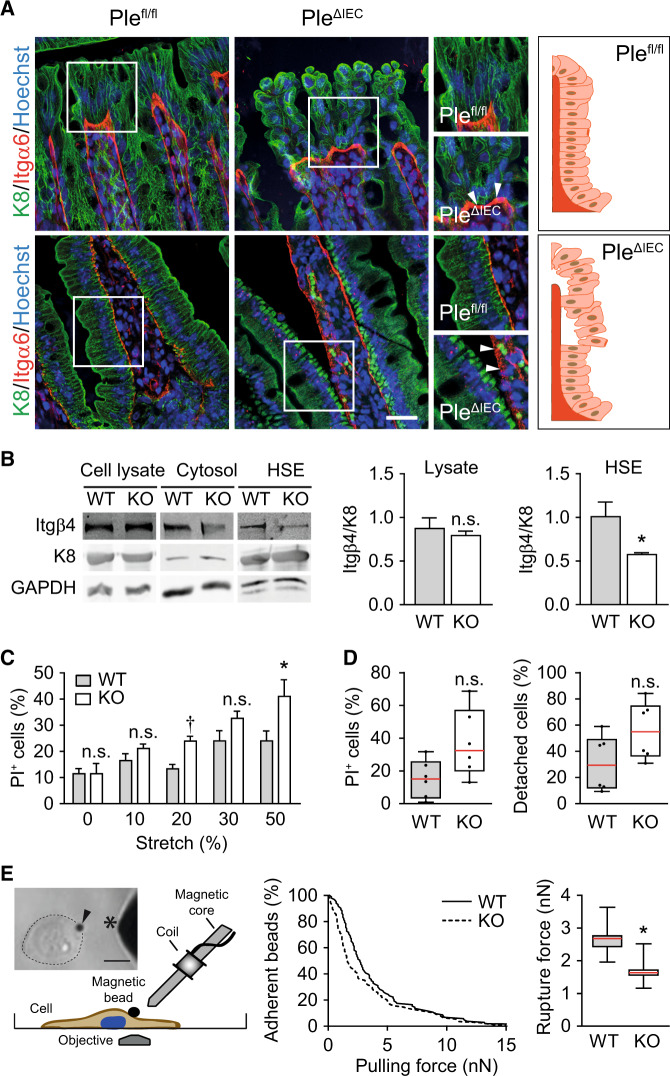
Plectin stabilizes IEC hemidesmosomes through KF recruitment. **A** Representative immunofluorescence images of *Ple*^*fl/fl*^ and *Ple*^*ΔIEC*^ distal colon (upper panels) and small intestine (lower panels) sections immunolabeled for K8 (green) and Itgα6 (red); Hoechst-stained nuclei (blue). Arrowheads, Itgα6-positive clusters. Scale bar, 25 μm. Boxed areas show ×1.5 images. Drawn schematics depict aligned, BM-attached *Ple*^*fl/fl*^ IECs (upper panel) and mislocalized, detaching *Ple*^*ΔIEC*^ IECs (lower panel). **B** Cell lysates, cytosol fractions, and keratin-enriched high salt extracts (HSE) were prepared from WT and KO Caco-2 cells and subjected to immunoblotting with antibodies to Itgβ4 and K8. GAPDH, loading control. Graphs show relative band intensities normalized to average *Ple*^*fl/fl*^ values, *n* = 4–6. **C** Viability of WT and KO Caco-2 cells exposed to uniaxial cyclic stretch presented as a percentage of dead (PI-positive; PI^+^) cells, *n* = 9–11. **D** Quantification of WT and KO Caco-2 cell viability (left) and adhesion (right) under radial shear flow shown as a percentage of dead and detached cells, respectively, *n* = 6. Boxplot data represent median, 25th, and 75th percentile with whiskers reaching the last data point. **E** Adhesion strength between ECM-coated superparamagnetic beads and WT and KO Caco-2 cells was quantified using magnetic tweezers that generated forces ramps at a speed of 1 nN/s up to a maximum force of 15 nN. Image and schematic depict magnetic tweezer setup. Arrowhead, paramagnetic bead; asterisk, magnetic tweezer tip; dotted circular line, cell border. Scale bar, 20 μm. The graph shows the percentage of beads (*n* = 103 WT, 109 KO cells) that remained adherent at a given pulling force. The boxplot shows the distribution of the median detachment force (calculated from bootstrapping by sampling with replacement, *n* = 1000 runs) and its distribution (25th, and 75th percentile with whiskers reaching the minimum and maximum sampled values). Bar graph data in all other subplots represent mean ± SEM, n.s. not significant, *P < 0.05, ^†^*P* < 0.001.

Plectin-mediated attachment of the keratin network to Itgα6β4-containing HDs plays a crucial role in stabilizing the adhesion of keratinocytes to the matrix and hence imparts mechanical stability to the skin^20,31^. To examine whether the *Ple*^*ΔIEC*^ intestine phenotypically follows the same paradigm, we scrutinized colon and small intestine sections immunolabeled for K8 and Itgα6 (Fig. 5A) or collagen (Col) IV (Fig. S7). In line with the observations from H&E- and Sirius red-stained colon sections (Figs. 2A and S7), *Ple*^*ΔIEC*^ IECs partially lost their polarized orientation (Fig. S8A–C); they were misaligned and largely detached from the BM at the luminal surface of the crypts (Fig. 5A, upper panels). Despite a partial loss of apicobasal polarity of *Ple*^*ΔIEC*^ IECs (Fig. S8A–C), the epithelium retained a characteristic polarized distribution of the apical markers villin and ezrin (Fig. S8D, E). The extensive detachment of *Ple*^*ΔIEC*^ IECs was even more apparent in the small intestine, where we often found the whole epithelial sheet physically separated from underlying structures (Fig. 5A, lower panels). Remarkably, in both the *Ple*^*ΔIEC*^ colon and the small intestine, Itgα6-positive patches remained confined to the BM, while detached IECs were entirely devoid of Itgα6 signals. Thus we conclude that plectin ablation abrogates the functional link between KFs and HDs.

To address the effects of plectin ablation biochemically, we prepared keratin-enriched cell fractions^19^ from human WT and KO IEC Caco-2 lines and compared their integrin (Itg) content by immunoblotting using antibodies to Itgβ4. As expected, such cell fractions were highly enriched in keratins 8 (Fig. 5B), 18, and 19 (not shown). Although Itgβ4 levels were comparable in cell lysates, the Itgβ4 content of insoluble keratin fractions was significantly reduced in KO cells compared to WT cells (Fig. 5B). These observations correlated well with the histological data (Fig. 5A).

To assess whether plectin deficiency affects the biomechanical properties of IECs, we performed a series of quantitative assays with human IEC lines Caco-2 and hCC. Monitoring cell viability under mechanical stress on a stretched flexible membrane (uniaxial cyclic stretch) revealed the higher mechanical vulnerability of both KO cell lines, as the proportion of PI-positive (dead) cells significantly increased with stretch amplitudes ranging from 10% to 50% (Figs. 5C and S9A). Reduced mechanical resistance of KO cells was confirmed by fluid shear stress assay using a spinning disc device. When exposed to constant radial flow, KO cells displayed death rates about twice as high as that of their WT counterparts (Figs. 5D and S9B). Moreover, the fraction of detached Caco-2 (but not hCC) cells was higher for KO than WT cells. This suggests that plectin ablation weakens their adhesion to the underlying substratum.

To confirm this hypothesis, we quantified adhesion strength between ECM-coated superparamagnetic beads and cell adhesions using magnetic tweezers. We applied increasing forces of up to 15 nN to magnetic beads (force increase at 1 nN/s) and recorded the force at which each bead detached from the cell. From a total of >100 detachment events for each cell type, we calculated the cumulative detachment probability as a function of pulling force and report the force at which 50% of the beads detached from the cells (Figs. 5E and S9C). We measured lower detachment forces in both Caco-2 and hCC KO compared to WT cells, which confirms our hypothesis of weaker Itg-mediated adhesions in plectin-deficient cells. Hence, like for skin type I HDs^20^, plectin loss is deleterious for the stability of type II HDs present in the intestine, leading to compromised mechanical resilience of IECs and intestinal epithelia.

### IEC-specific plectin deficiency exacerbates experimental colitis

**Fig. 6:**
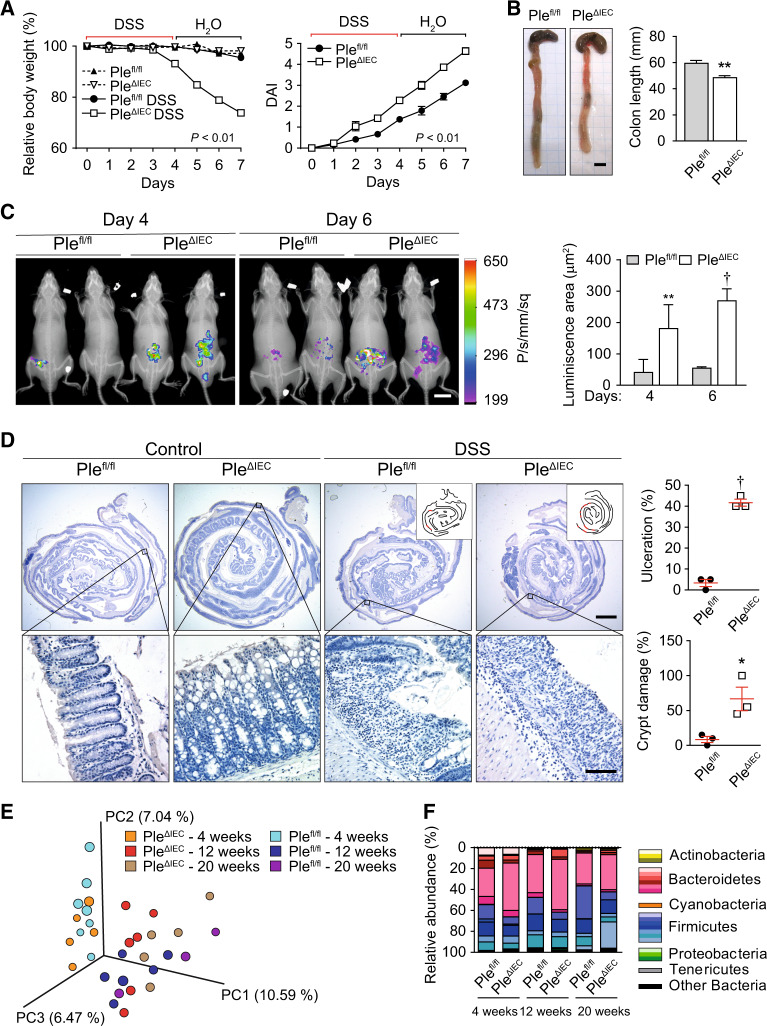
*Ple*^*ΔIEC*^ mice are more susceptible to DSS-induced colitis. **A** Relative bodyweight and disease activity index (DAI) of untreated and DSS-treated *Ple*^*fl/fl*^ and *Ple*^*ΔIEC*^ mice during experimental colitis. Four to seven mice per genotype and time point were analyzed. **B** Representative images of colon and caecum of DSS-treated *Ple*^*fl/fl*^ and *Ple*^*ΔIEC*^ mice. The graph shows colon length, *n* = 4–6. **C** In vivo chemiluminescence images and signal quantification (graph) of DSS-treated *Ple*^*fl/fl*^ and *Ple*^*ΔIEC*^ mice injected with the myeloperoxidase substrate luminol on days 4 and 6 of DSS treatment, *n* = 3–4. **D** Representative hematoxylin-stained sections of Swiss roll mounts from untreated (control) and DSS-treated (DSS) mice. Scale bars, 2 mm, magnified boxed areas, 100 μm. Insets, outlines of lesions (in red) distributed along mucosa (black lines) in corresponding panels. Graphs show quantification of colonic tissue damage given as the percentage of ulceration and crypt damage, *n* = 3, **E**, **F** Fecal microbiota beta diversity in 4-, 12-, and 20-week-old untreated *Ple*^*fl/fl*^ and *Ple*^*ΔIEC*^ mice as determined by 16S rDNA sequencing. Principal coordinate analysis plot (**E**), constructed with unweighted UniFrac distance metric, shows clustering of microbial beta diversity. PC1, PC2, and PC3 represent the top three principal coordinates that captured most of the diversity (given as a percentage). Global composition (**F**) of bacterial microbiota at phyla level shown as relative operational taxonomic unit (OTUs) abundance per time point and genotype, *n* = 4–6. Data are presented as mean ± SEM, *P < 0.05, **P < 0.01, †*P* < 0.001.

The spontaneous colitic phenotype in *Ple*^*ΔIEC*^ mice (Fig. 1) suggests that plectin deletion can contribute substantially to the pathogenesis of UC. To assess whether loss of plectin increases the susceptibility to colitis, we induced experimental colitis in *Ple*^*fl/fl*^ and *Ple*^*ΔIEC*^ mice. Even a short exposure (3–4 days) to low DSS doses (1.5–2%) resulted in a dramatic bodyweight loss of *Ple*^*ΔIEC*^ mice, in sharp contrast to similarly treated *Ple*^*fl/fl*^ which experienced only insignificant weight losses (Fig. 6A). The weight loss of mutant mice was associated with a higher disease activity index (DAI; Fig. 6A), a decreased survival rate (not shown), and a significant reduction in colon length (Fig. 6B). The severity of induced colitis in *Ple*^*ΔIEC*^ mice coincided with larger inflamed areas at days 4 and 6 after the initiation of DSS-treatment (Fig. 6C), corresponding to a higher influx of MPO-positive neutrophils and intestinal epithelial injury. Further, histological evaluation of “Swiss rolls” of the entire colon confirmed these results and revealed clear signs of inflammation and epithelial damage in all DSS-treated animals. However, large regions with heavy ulceration, crypt damage, and inflammatory response in *Ple*^*ΔIEC*^ mice were in striking contrast to fewer lesions in *Ple*^*fl/fl*^ mice (Figs. 6D and S10A).

Since gut microbial dysbiosis is a typical finding in UC patients^32–34^, we compared the composition of fecal microbiota in unchallenged *Ple*^*fl/fl*^ and *Ple*^*ΔIEC*^ mice at the ages of 4, 12, and 20 weeks. Surprisingly, despite the impaired intestinal barrier and concomitant inflammation phenotype of *Ple*^*ΔIEC*^ mice (Fig. 1F–L), we observed no significant differences in alpha (Fig. S10B) and beta (Fig. 6E) diversities between both genotypes. In all animals, bacterial microbiota was dominated by bacteria belonging to families S24-7 (bacteroidetes), lactobacillaceae (firmicutes), and lachnospiraceae (firmicutes) (Fig. 6F). Together, these data show higher susceptibility of *Ple*^*ΔIEC*^ mice to DSS-induced colitis, accompanied by severe epithelial damage and inflammation in the absence of microbial dysbiosis.

### Reduced mechanical stability of epithelia accounts for intestinal injury in *Ple*^*ΔIEC*^ mice

To identify the onset and time course of intestinal injury in *Ple*^*ΔIEC*^ mice, we assessed intestinal epithelial damage scores in newborn, 21-day-old, and 12-week-old mice (Figs. S11A and 7A–C). While newborn mice were histologically inconspicuous, the colon and the small intestine displayed first signs of damage in 21-day-old *Ple*^*ΔIEC*^ mice (see also Fig. S12B), which coincided with weaning and transition to solid chow. The onset of the epithelial breach was accompanied by the subsequent development of inflammatory response as shown by increased immune cell infiltration, extent (or intensity) of inflammation, and lymphatic follicle number/size (Fig. S12A).

**Fig. 7:**
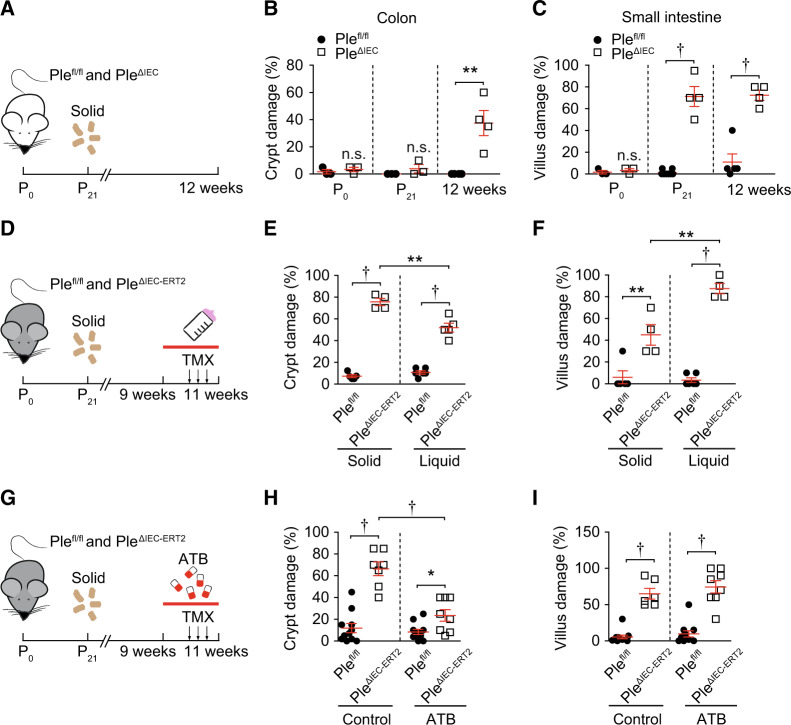
Intestinal epithelial damage in *Ple*^*ΔIEC*^ mice results from mechanical stress. **A**–**C** *Ple*^*fl/fl*^ and *Ple*^*ΔIEC*^ mice were sacrificed on postnatal day 0 (P0), postnatal day 21 (P21), and at 12 weeks (12w) of age, and epithelial damage scores were assessed from colon and small intestine sections. Schematic illustrates the experimental setup (**A**). Solid, transition to solid chow at P21. Graphs show quantification of epithelial damage in the colon (**B**) and small intestine (**C**) at the age indicated. **D**–**F** Nine-week-old *Ple*^*fl/fl*^ and *Ple*^*ΔIEC- ERT2*^ mice were either kept on solid chow or provided with a liquid diet for 14 days. *Plectin* inactivation was induced by three consecutive applications of tamoxifen (TMX) on days 6, 8, and 10; mice were sacrificed on day 14. The schematic illustrates the experimental setup (**D**). Solid, transition to solid chow at P21; arrows, TMX application; red bar, period on a liquid diet. Graphs show quantification of epithelial damage in the colon (**E**) and small intestine (**F**) on solid chow and liquid diet. **G**–**I** Nine-week-old *Ple*^*fl/fl*^ and *Ple*^*ΔIEC-ERT2*^ mice were kept either untreated or treated with broad-spectrum antibiotics. *Plectin* inactivation and sample collection were identical to (**B**). Schematics illustrate experimental setup (**G**). Chow, the transition to solid chow at P21; arrows, TMX application; red bar, period of antibiotics (ATB) treatment. Graphs show quantification of epithelial damage in the colon (**H**) and small intestine (**I**) on solid chow and liquid diet. Data are presented as mean ± SEM, n.s. not significant, *P < 0.05, **P < 0.01, ^†^*P* < 0.001.

To gain better control over *plectin* inactivation timing, we generated TMX-inducible IEC-specific *plectin* knockout (*Ple*^*ΔIEC-ERT2*^) mice. Three consecutive applications of TMX in 9-week-old *Ple*^*ΔIEC-ERT2*^ mice resulted in recombination efficiency comparable to that of constitutive *Ple*^*ΔIEC*^ mice, and a distinctive intestinal phenotype developed as early as 5 days post-TMX administration (not shown). To determine the effect of a diet change on the intestinal injury, *Ple*^*ΔIEC-ERT2*^ and *Ple*^*fl/fl*^ control mice were either kept on solid chow or provided with a low-residue liquid diet 6 days before TMX administration. The liquid diet significantly attenuated epithelial damage in the colon of *Ple*^*ΔIEC-ERT2*^ mice; however, the histological score indicates more severe injury of the small intestine (Figs. 7D–F and S11B). The beneficial effects of the liquid diet also manifested as less prominent colon swelling (not shown).

As previous studies linked the severity of colitis and intestinal injury with commensal microbiota^8,35^, next we treated *Ple*^*fl/fl*^ and *Ple*^*ΔIEC-ERT2*^ mice with well-established broad-spectrum antibiotics^8^. In our experimental setup, the apparent milder colitic phenotype consistently coincided with lower epithelial damage in the colon of *Ple*^*ΔIEC-ERT2*^ mice. This treatment however did not affect epithelial injury of the *Ple*^*ΔIEC-ERT2*^ small intestine (Figs. 7G–I and  S11C). Collectively, these data support the notion that the increased susceptibility of the plectin-deficient intestinal epithelium to mechanical strain impinged by luminal content is caused by a lack of KF attachment to Itg clusters and destabilization of intestinal HDs. Further, the fact that antibiotics also partially alleviate epithelial damage suggests that luminal bacteria significantly contribute to intestinal injury in *Ple*^*ΔIEC-ERT2*^ mice.

## Discussion

The intestinal epithelium faces substantial mechanical stress^36^, which is inextricably linked to gut physiology. Although several studies suggest the importance of intestinal KF networks^35,37,38^ and KF-associated cell junctions (Ds and HDs)^8,9^ for protection against intestinal inflammation and CRC, the contribution of altered epithelial mechanics to observed phenotypes remain unexplored. Here, we focus on the role of KF-cell junction linker plectin in the maintenance of intestinal homeostasis, and we provide a comprehensive analysis of molecular mechanisms governing the mechanical stability of intestinal epithelia.

The most notable phenotype of both plectin-deficient mouse models (*Ple*^*ΔIEC*^ and *Ple*^*ΔIEC-ERT2*^) is the detachment of IECs from the underlying BM, resulting in extensive epithelial injury and eventually in the spontaneous development of a colitic phenotype. Strikingly, this is accompanied by loss of the hemidesmosomal ECM receptor Itgα6 from detached IECs, while Itgα6 patches remain on a collagen-stained BM, likely indicating their inefficient linkage to cytoskeletal structures. Indeed, our TEM analysis revealed that less electrodense HDs formed by *Ple*^*ΔIEC*^ IECs were somewhat elongated, and gaps between HD plaques and the BM were significantly wider compared to *Ple*^*fl/fl*^ IECs. The formation of morphologically abnormal HDs was paralleled with reduced expression levels of both HD-forming integrins (α6 and β4) in the *Ple*^*ΔIEC*^ mucosa. Moreover, the content of Itgβ4 was also significantly diminished in keratin-enriched fractions prepared from plectin-deficient IECs, suggesting the reduced association of Itgα6β4 complexes with intestinal keratins (K8, 18, and 19).

Our observations are concordant with a recently published model for skin type I HD^10^, which proposed that plectin (along with BPAG, another plakin family member) fortifies HD plaques both horizontally (by a lateral association of Itgβ4) and vertically (by interlinking Itgβ4 with KFs). Accordingly, ablation of plectin, the only plakin present in intestinal-type II HD^39^, would fully abrogate a functional link between KFs and HDs and would result in their overall destabilization. In line with this hypothesis, we in vitro show a trend towards higher detachment (paralleled with a higher death rate) of plectin-deficient IECs exposed to a uniaxial cyclic stretch and a constant radial flow compared to their WT counterparts. As both *Ple*^*fl/fl*^ and *Ple*^*ΔIEC*^ IECs display a minimal degree of spontaneous apoptosis in vivo, the observed excessive cell death in our experiments in vitro can likely be attributed to incomparable force magnitude and cell context under these two conditions. Consistent with our results from cell stretching and radial shear assays, we also determine significantly lower adhesion strength between ECM-coated superparamagnetic beads and plectin-deficient IECs using magnetic tweezers. Hence, by combining in vivo and in vitro approaches, we provide evidence that plectin is essential for the stability of intestinal HD type II, a structure preventing colitis^8^ and presumably also the risk of colitis-associated CRC^8,40,41^.

Previous studies demonstrated that the deletion of *plectin* has adverse effects on the formation of intercellular junctions, with consequences for the epithelial barrier function^21,22^. It has been shown that plectin-deficient cholangiocytes form dysfunctional Ds and fail to upregulate some desmosomal proteins, such as desmoplakin, a putative binding partner of plectin^42^, in response to bile stasis^22^. This failure results in mechanical weakening of the biliary epithelium and contributes to plectin-related familial intrahepatic cholestasis^43^. In terms of mechanistic parallels between plectin-deficient biliary and intestinal epithelia, we found that apart from destabilizing HDs, plectin deficiency also leads to the prominent broadening of Ds, AJs, and TJs. Moreover, *Ple*^*ΔIEC*^ IECs exhibit downregulation of corresponding junctional constituents (ZO-1, E-cad, Dsg2, and Dsp1/2). Although the resulting dilatation of intercellular spaces would per se suffice to explain the observed increase in intestinal permeability and bacterial penetration, the “leaky gut” in *Ple*^*ΔIEC*^ mice seems ultimately rooted in the less firm IEC/BM connection, given the extent of IEC detachment. On the other hand, proper anchorage of KFs (determining cell mechanics) to Ds (ensuring intercellular cohesion) is known to provide load-bearing tissues with mechanical stability^11^. Showing altered KF cytoarchitecture and aberrant D formation in both in vitro plectin-deficient IEC systems and *Ple*^*ΔIEC*^ mice, our results suggest that D-keratin complex abnormality substantially contributes to the compromised mechanics of the *Ple*^*ΔIEC*^ intestinal epithelium.

We propose that the lack of functional plectin at HDs (in combination with its effects on KFs and Ds) and the resulting mechanical epithelial fragility favor an impaired intestinal barrier function and are ultimately responsible for colitis. Importantly, comparable mucosal deterioration was observed upon plectin ablation during development and in the adult intestine with its fully mature immune system. Furthermore, we also demonstrate that loss of plectin can exacerbate experimental colitis in mice. Consistent with these results, lower expression levels of plectin correlate with UC development in human patients, suggesting that defects in cytoskeleton coordination mediated through plectin contribute to IBD pathogenesis in humans by affecting IEC/BM adhesion, IEC cohesion, and mechanical properties. However, it is well recognized that properly organized KF networks^38,44^, HDs^8^, and intercellular junctions^2,3,9^ exert numerous non-mechanical functions, providing the intestinal epithelium with protection against microbial infection and uncontrolled inflammation. Our data do not rule out similar functions in the *Ple*^*ΔIEC*^ intestine. Further studies will be required to investigate how plectin deficiency affects cell-autonomous (barrier function-independent) mechanisms involved in the interplay between IECs, gut microbiota, and immune cells.

We observed a prominent hyperproliferation of plectin-deficient IECs paralleled by a dramatically higher proportion of PAS-positive goblet cells. This phenotype closely resembles the situation in mice lacking hemidesmosomal α6 integrin^8^ or K8^45,46^. Together, these findings suggest that the keratin/plectin/integrin axis is essential for balanced proliferation and differentiation of IECs. Interestingly, intestinal K8 and K18 were shown to promote Notch1 signaling, a major pathway of colonic cell fate specification^46^. Since Notch-mediated signal transduction depends on cytoskeletal tension^47,48^, it is tempting to speculate that intact plectin-controlled KF cytoarchitecture facilitates mechano-regulation of intestinal cell fate. Moreover, plectin anchors the cytoskeleton to the nuclear envelope via interaction with nesprin-3^49^ and mediates transmission of mechanical stimuli directly to the nucleus. Multiple studies provide evidence that loss of plectin results in nuclear phenotypes, including altered nuclear positioning^22^, nuclear deformations^50,51^, chromatin modification, and gene expression^51^. To elucidate whether and how plectin regulates specific transcriptional programs in IECs and to find their contribution to the proper spatiotemporal proliferation/differentiation pattern within colonic crypts is a goal of our ongoing studies.

The rapid deterioration of the *Ple*^*ΔIEC*^ intestinal mucosa following weaning (i.e., a switch to a solid diet and the amplification of muscle contractions) indicates that the origin of colitis in the absence of plectin is primarily associated with a reduced capacity of IECs to resist mechanical stress. In addition, the most severe epithelial injury was found in the distal colon, which is the region most intensely subjected to such stress. Comparably devastating epithelial instability has been well documented for epidermal layers in EB patients^15,16^. As there is no causal therapy for EB available^15^, the current treatment focuses mainly on the prevention of tissue destruction. Following the same rationale, we demonstrate that a low-residue liquid diet significantly attenuates colonic epithelial damage, thus protecting its barrier function. Surprisingly, this approach aggravates IEC detachment in the *Ple*^*ΔIEC*^ small intestine, which might suggest augmented susceptibility of the small intestine to plectin loss. Therefore, future studies should investigate whether differential expression of plectin along the gastrointestinal tract might have an impact on regional differences in disease manifestations in patients. In line with the previous observations^8,35^, antibiotic treatments markedly decrease not only mucosal inflammation but, intriguingly, also epithelial damage, which implies that host-microbiota interactions contribute to excessive IEC sloughing in the *Ple*^*ΔIEC*^ intestine. Although dietary factors can likely ameliorate only less extensive trauma, our results suggest that a low-residue liquid diet combined with antibiotic treatment might be a useful palliative modality. To translate our findings into clinical medicine: it remains to be determined whether such a strategy (i) is suitable for long-term treatment and (ii) is effective with respect to systemic disease manifestation.

## Methods

### Patients

Colon biopsy samples were collected from patients diagnosed with UC (*n* = 97) and from healthy controls (*n* = 20) admitted to the Hepatogastroenterology Department at the Institute for Clinical and Experimental Medicine (Prague, Czech Republic) for a colonoscopy from July 2016 to May 2019. Subjects were assigned to the healthy control group only after all clinical examinations excluded any signs of autoimmune disease, inflammatory disease, and colon cancer. All UC patients with concurrent primary sclerosing cholangitis (PSC) were excluded from the study. Endoscopic UC activity at the time of a standard optical colonoscopy was categorized according to the Mayo endoscopic subscore and confirmed by histology examinations of the grade of inflammation. Clinical characteristics of patients are shown in Supplementary Table S1. Standard endoscopic biopsies were extracted from the inflamed non-dysplastic mucosa of the left colon (rectum) and immediately placed in an RNAlater solution. Total RNA was extracted according to the manufacturer’s instructions.

### Mice

*Plectin*^*flox/flox*^ (*Ple*^*fl/fl*^) mice^23^ were crossed with *villin-Cre* transgenic mice (MGI 2448639) to generate *Ple*^*fl/fl*^/*villin-Cre* mice (*Ple*^*ΔIEC*^) and with *villin-creERT2* transgenic mice (MGI 3053826; both Cre strains were kindly provided by S. Robine^52^) to generate *Ple*^*fl/fl*^/*villin-creERT2* mice (*Ple*^*ΔIEC-ERT2*^). Age-matched littermate male mice were used in all experiments. Unless stated otherwise, mice were 12–14 weeks old. Animals were housed under specific pathogen-free conditions with regular access to chow and drinking water and a 12 h light/12 h dark regime.

### Cells and CRISPR-mediated targeting of plectin

Caco-2 cells were grown in Dulbecco’s modified Eagle medium (DMEM) supplemented with 20% fetal bovine serum (FBS) in a 5% CO_2_/air humidified atmosphere at 37 °C. Human colonic cells (hCC; T0570, Applied biological materials, Inc.) were cultured in DMEM supplemented with 10% FBS in 5% CO_2_/air humidified atmosphere at 37 °C. Plectin knockout (KO) cell lines were generated by targeting genomic sequences of intron 25–26 and exon 31 of *Plectin* using CRISPR/Cas9 plasmid pX330 Cas9-Venus (a kind gift of B. Schuster, IMG CAS, Prague, Czech Republic) as described previously^22^. The potential off-target sites were predicted using CRISPOR (http://crispor.tefor.net/). The four top-ranking potential off-target sites for each guide RNA were selected for validation. The genomic DNA sequences surrounding the potential off-target sites were amplified by PCR using gene-specific primers (Supplementary Table 3). PCR products were analyzed by direct sequencing (Figs. S12 and S13).

### Statistics

All results are presented as mean ± SEM. All normally distributed parametric data were analyzed by two-tailed unpaired Student *t* test. Comparisons of multiple groups to controls were performed using two-tailed one-way ANOVA. Comparisons of frequency distributions of BrdU-positive cells were analyzed with Mann–Whitney test. Survival curves were analyzed by Mantel–Cox test. Statistical analyses were performed using GraphPad Prism 5 (GraphPad Software, Inc., La Jolla, CA). Comparisons of detachment forces were done with bootstrapping (sampling with replacement) with 1000 replicates. Statistical significance was determined at the levels of *P < 0.05, **P < 0.01, ^†^*P* < 0.001; n values are specified in the figure legends.

### Study approval

This study was approved by the Ethics Committee of the Institute for Clinical and Experimental Medicine and Thomayer Hospital with Multi-Center Competence (G16-06-25). Written informed consent was obtained from all subjects before the study. All animal studies were performed in accordance with European Directive 2010/63/EU and were approved by the Czech Central Commission for Animal Welfare (48/2014 and 23/2020).

## Supplementary information

supplementary information
