# Correction: Plectin ensures intestinal epithelial integrity and protects colon against colitis

**DOI:** 10.1038/s41385-021-00463-x

**Published:** 2021-10-22

**Authors:** Alzbeta Krausova, Petra Buresova, Lenka Sarnova, Gizem Oyman-Eyrilmez, Jozef Skarda, Pavel Wohl, Lukas Bajer, Eva Sticova, Lenka Bartonova, Jiri Pacha, Gizela Koubkova, Jan Prochazka, Marina Spörrer, Christopher Dürrbeck, Zuzana Stehlikova, Martin Vit, Natalia Ziolkowska, Radislav Sedlacek, Daniel Jirak, Miloslav Kverka, Gerhard Wiche, Ben Fabry, Vladimir Korinek, Martin Gregor

**Affiliations:** 1 Laboratory of Integrative Biology, Institute of Molecular Genetics of the Czech Academy of Sciences, Prague, Czech Republic; 2 Department of Clinical and Molecular Pathology, Faculty of Medicine and Dentistry, Palacky University and University Hospital in Olomouc, Olomouc, Czech Republic; 3 Institute of Pathology, University Hospital Ostrava, Ostrava, Czech Republic; 4 Department of Gastroenterology and Hepatology, Institute for Clinical and Experimental Medicine, Prague, Czech Republic; 5 Department of Clinical and Transplant Pathology, Institute for Clinical and Experimental Medicine, Prague, Czech Republic; 6 Department of Pathology, Third Faculty of Medicine, Charles University, Prague, Czech Republic; 7 Department of Epithelial Physiology, Institute of Physiology of the Czech Academy of Sciences, Prague, Czech Republic; 8 Czech Centre for Phenogenomics, Institute of Molecular Genetics of the Czech Academy of Sciences, Prague, Czech Republic; 9 Laboratory of Transgenic Models of Diseases, Institute of Molecular Genetics of the Czech Academy of Sciences, Prague, Czech Republic; 10 Department of Physics, University of Erlangen-Nuremberg, Erlangen, Germany; 11 Laboratory of Cellular and Molecular Immunology, Institute of Microbiology of the Czech Academy of Sciences, Prague, Czech Republic; 12 Faculty of Mechatronics Informatics and Interdisciplinary Studies, University of Liberec, Liberec, Czech Republic; 13 Institute of Biophysics and Informatics, First Faculty of Medicine, Charles University, Prague, Czech Republic; 14 Department of Radiodiagnostic and Interventional Radiology, Institute for Clinical and Experimental Medicine, Prague, Czech Republic; 15 Department of Biochemistry and Cell Biology, Max F. Perutz Laboratories, University of Vienna, Vienna, Austria; 16 Laboratory of Cell and Developmental Biology, Institute of Molecular Genetics of the Czech Academy of Sciences, Prague, Czech Republic

Correction to: *Mucosal Immunology* 10.1038/s41385-021-00380-z, published online 05 March 2021

Informations about authors in the head of the article and affiliations section:

The afiliation of the co-author Daniel Jirak is currently incorrectly listed as University of Liberec, Faculty of Mechatronics Informatics and Interdisciplinary Studies, Liberec, Czech Republic. It shoul be Technical University of Liberec, Faculty of Health Studie, Liberec, Czech Republic.

