# Supplementary material for: Plectin ensures intestinal epithelial integrity and protects colon against colitis
Source: Mucosal Immunol. 2021 Mar 5;14(3):691–702. doi: 10.1038/s41385-021-00380-z (PMC8076044; doi:10.1038/s41385-021-00380-z)
Supplement: Supplementary file 1 — supplementary information [file 41385_2021_380_MOESM1_ESM.pdf]

SUPPLEMENTARY MATERIAL

Plectin Ensures Intestinal Epithelial Integrity and Protects Colon Against Colitis

Alzbeta Krausova, Petra Buresova, Lenka Sarnova, Gizem Oyman-Eyrilmez, Jozef Skarda, Pavel Wohl, Lukas Bajer, Eva Sticova, Lenka Bartonova, Jiri Pacha, Gizela Koubkova, Jan Prochazka, Marina Spörrer, Christopher Dürrbeck, Zuzana Stehlikova, Martin Vit, Natalia Ziolkowska, Radislav Sedlacek, Daniel Jirak, Miloslav Kverka, Gerhard Wiche, Ben Fabry, Vladimir Korinek, Martin Gregor

Correspondence to:

Martin Gregor, PhD

Laboratory of Integrative Biology

Institute of Molecular Genetics of the Czech Academy of Sciences

Videnska 1083, Prague 4, CZ-14220, Czech Republic

Tel: +420 241 063 461; Fax: +420 224 310 955

Email: martin.gregor@img.cas.cz

1. Supplementary METHODS

2. Supplementary TABLES

3. Supplementary REFERENCES

4. Supplementary FIGURES

1. Supplementary METHODS

DSS-induced colitis and disease activity scoring. 12-week-old *Ple*$^{fl/fl}$ and *Ple*$^{\Delta IEC}$ mice were provided with a 2% DSS (TdB Consultancy) dissolved in drinking water *ad libitum* over 4 days. Then they were provided with drinking water over 3 days. Mice were sacrificed on day 8. As described previously[1], body weight, stool consistency, and rectal bleeding (Hemoccult Fecal Occult Blood Test, Beckman Coulter) were assessed daily to calculate the disease activity index (DAI).

Depletion of gut microbiota by antibiotic treatment. Streptomycin 2 g/l (Carl Roth), gentamycin 200 mg/l (Carl Roth), enrofloxacin 100 mg/l (Sigma-Aldrich), and bacitracin 1 g/l (Carl Roth) were provided to 9-week-old *Ple*$^{fl/fl}$ and *Ple*$^{\Delta IEC\ ERT2}$ mice in drinking water *ad libitum* over 2 weeks. To induce specific plectin deletion in 9-week-old *Ple*$^{\Delta IEC\ ERT2}$ mice, 5 mg of tamoxifen (Sigma-Aldrich; dissolved in 200 µl of sunflower oil) was administered twice a day on days 6, 8, and 10 of the antibiotic treatment by orogastric gavage. The stool was collected before and after the treatment, and its microbial content was analyzed. Mice were sacrificed on day 14 of the treatment. *Ple*$^{fl/fl}$ mice received sunflower oil only.

Liquid diet feeding. 9-week-old *Ple*$^{fl/fl}$ and *Ple*$^{\Delta IEC\ ERT2}$ mice received a low-residue Ensure Plus (Abbott Laboratories) nutritional supplement diluted 1:1 in drinking water in the course of 2 weeks *ad libitum* in the absence of solid chow. Water was offered *ad libitum*. 5 mg of tamoxifen (Sigma-Aldrich; dissolved in 200 µl of sunflower oil) was administered twice a day on days 6, 8, and 10 of the liquid diet by orogastric gavage. Mice were sacrificed on day 14 of the liquid diet. Control mice received solid chow over the same time period. *Ple*$^{fl/fl}$ mice received sunflower oil only.

BrdU incorporation assay. 14-week-old *Ple*$^{fl/fl}$ and *Ple*$^{\Delta IEC}$ mice received an intraperitoneal injection of 50 mg/kg 5-bromo-2'-deoxyuridine (BrdU; Sigma-Aldrich). Mice were sacrificed 2, 24, and 48 hours after the injection. The small intestine and the colon were dissected and processed for immunohistochemistry (see below). BrdU-positive cells were visualized by anti-BrdU antibody (BMC9318, Roche).

Whole-body imaging of inflammation. Myeloperoxidase (MPO) activity was detected by *in vivo* whole-body imaging using a specific XenoLight RediJect Chemiluminescent Inflammation Probe (PerkinElmer). A freshly thawed probe solution was administered by an intraperitoneal injection (200 mg/kg). Mice were immediately moved into the imaging chamber (Xtreme – whole-body imaging system, Bruker), and the number of photons produced by an inflammation probe was recorded (10 min after its administration) in anesthetized mice with 5 min exposure time.

Histology, immunohistochemistry, and immunofluorescence. Formalin-fixed and paraffin-embedded small intestine (ileum) and colon sections (5 μm) were stained with haematoxylin and eosin (H&E; Sigma-Aldrich), Sirius Red[2], Alcian blue, nuclear fast red (NRF), and the periodic acid-Schiff stain (Sigma-Aldrich) according to the manufacturer's protocol. For immunohistochemistry and immunofluorescence, paraffin sections were subjected to heat-induced antigen retrieval in either Tris-EDTA (pH 9) or citrate (pH 6) buffer supplemented with Tween 20 and further permeabilized with 0.1 M glycine and 0.1% Triton X-100 for 15 min. To block endogenous peroxidase activity and non-specific antigen interactions, sections were incubated with 0.3% hydrogen peroxide for 15 min followed by 5% bovine serum albumin (BSA; Sigma-Aldrich) in PBS supplemented with 0.1% Tween 20 (PBST) for 1 hour. Afterwards, sections were incubated with primary antibodies at 4°C overnight, followed by incubation with horseradish peroxidase- (HRP) or fluorophore-conjugated secondary antibodies at room temperature for 1 hour. The HRP signal was visualized with 3,3'-diaminobenzidine (DAB) detection kit (Roche). Caco-2 cells seeded on coverslips were fixed with ice-cold methanol for 1 min. Non-specific antigen interactions were blocked with 5% BSA in PBST for 1 hour. Incubation with primary antibodies followed by incubation with secondary antibodies was performed at room temperature for 1 hour. The following primary antibodies were used: Ki-67 (GTX16667, GeneTex), keratin 8 (Troma I, Developmental Studies Hybridoma Bank), collagen IV (2150-1470, BioRad), pan-keratin (z0622, Agilent/Dako), E-cadherin (610181, BD Biosciences), desmoglein (611002, Progen), desmoplakin (651109, Progen), integrin α6 (ab181551, Abcam), plectin (GP21, Progen), chromogranin A (ab15160, Progen), keratin 20 (clone Ks20.8, Agilent/Dako), keratin 19 (Troma III, Developmental Studies

Hybridoma Bank), β-actin (A2066, Sigma-Aldrich), villin (sc-58897, Santa Cruz), ezrin (ab4069, Abcam), and lysozyme (A0099, Agilent/DAKO). The following secondary antibodies were used: donkey anti-guinea pig Alexa Fluor (AF) 488, donkey anti-guinea pig AF594, donkey anti-rabbit AF488, goat anti-rabbit AF594, goat anti-rabbit HRP-conjugated, donkey anti-mouse AF488, donkey anti-mouse RhodamineRedX, donkey anti-rat AlexaFluor488, goat anti-mouse HRP-conjugated (all from Jackson ImmunoResearch). Nuclei were counterstained with Hoechst 33258 (Sigma-Aldrich). HRP-conjugated antibodies were visualized by DAB using the DAB Substrate (Roche Diagnostics) and counterstained with Mayer's hematoxylin (Sigma-Aldrich).

Visualization of Muc2 in colonic whole mounts. Staining was performed as described before[1]. Briefly, the intact colon was immediately fixed in Carnoy's fixative, and cut-open samples (5 x 8 mm) were incubated with an anti-Muc2 antibody (sc-15334, Abcam), at 4°C overnight followed by 1.5 hour-incubation with fluorescently labeled secondary antibodies (donkey anti-rabbit AF488, Jackson ImmunoResearch) at room temperature. Nuclei were counterstained with Hoechst 33258 (Sigma-Aldrich). Samples were placed into 100% glycerol and immediately visualized using a Leica TCS SP8 confocal microscope.

TUNEL assay. Apoptotic cells were visualized on paraffin-embedded tissue sections using a Click-iT TUNEL Alexa Fluor 488 kit (ThermoFisher Scientific) according to the manufacturer's instructions.

Image acquisition and processing. Immunofluorescence images were acquired using a Leica TCS SP8 confocal fluorescence microscope (Leica Microsystems) with HC PL FLUOTAR 25×/0.75 NA and HC PL APO 63×/1.4 NA immersion oil objectives. For tissue sections, z-stacks were acquired, and representative maximal projections were shown. Super-resolution microscopy was performed using either a DeltaVision OMX microscope with a Blaze SIM module (GE Healthcare Life Sciences) and an U APO N 100×/1.49 NA immersion oil objective (K8 staining) or a Leica TCS SP8 STED 3X microscope (Leica Microsystems) with an HC PL APO 100×/1.4 NA immersion oil objective (actin and tubulin staining). Raw images were deconvolved using Huygens Essential 4.0.0 software (Huygens;

Scientific Volume Imaging). Representative maximal projections of z-stacks are shown. Bright-field images were acquired on a Leica DM6000 wide-field microscope with an HC PLAN APO 20×/0.7 NA dry objective. Post-acquisition processing was performed with Photoshop CS6 (Adobe Systems Inc., Mountain View, CA) and the open-source Fiji image processing package[3].

Transmission electron microscopy. Immediately after a dissection, the distal colon free of feces was cut into small pieces (3x3 mm) and fixed in 2.5% glutaraldehyde in Sorensen's buffer. Samples were post-fixed with 1% OsO_4 in Sorensen's buffer, contrasted with 1% uranyl acetate in 50% ethanol overnight, dehydrated through a graded ethanol series followed by propylene oxide, and embedded into an Epon 812 substitute and Durkupan ACM (Sigma-Aldrich). Polymerized blocks were cut into 80-nm-thin sections, contrasted with an aqueous solution of uranyl acetate and inspected using a Morgagni 268 transmission electron microscope operated at 80 kV. Images were captured using a Mega View III CCD camera (Olympus Soft Imaging Solutions).

Histological and morphometric analyses. Blinded histopathology evaluation of human colon biopsy samples stained with H&E was independently performed by two trained pathologist (E.S. and L.B.). Blinded histopathology evaluation of mouse colon sections stained with H&E and PAS was performed by a trained pathologist (J.S.). The numbers of Ki-67-, PAS-, ChgA-, K20-, lysozyme-, and TUNEL-positive cells, and the numbers of detached cells per crypt (colon) or villus (small intestine) were counted and normalized to the total numbers of IECs. At least 7 crypts or villi were analyzed per mouse and genotype. Crypt and villus damage was assessed as a percentage of crypts/villi with >10% of IECs detached from the BM. To analyze migration of IECs in the colonic crypt, the relative position of each BrdU-positive cell within the crypt was assessed. For scoring the cell position, cells were numbered sequentially from the crypt base to lumen, with cell position 0 being occupied by the first cell at the base of each crypt. The Muc2-positive area was measured and quantified using Fiji software. At least 8 images were analyzed per genotype. The morphometry of IEC (the largest and the smallest orthogonal diameter) was evaluated from images immunolabeled with anti-K8 and anti-Itgα6 antibodies using Fiji software. At least 250 IEC were assessed in 3 mice per genotype.

In vivo intestinal permeability assay. To measure intestinal permeability, FITC-dextran 4 (4000 MW; TdB Consultancy) dissolved in PBS was administered by oral gavage (0.6 g/kg body weight) to mice after a 4-hour fast. Blood was obtained by retro-orbital bleeding from anesthetized mice 4 hours later and collected in heparin-coated tubes (Microvette® CB 300, Sarstedt). Then plasma was separated. Serum-FITC levels were measured at 488 nm using an Envision 2104 MultiLabel Reader (PerkinElmer).

Ex vivo intestinal transepithelial electrical resistance measurement. The Ussing chamber technique was used to measure intestinal transepithelial electrical resistance measurement (TEER). Whole-thickness segments of the proximal and distal colon were mounted in Ussing chambers (exposed area 0.096 cm^2) filled with a Krebs-Ringer solution containing (in mM) Na^+ (140.5), K^+ (5.4), Ca^{2+} (1.2), Mg^{2+} (1.2), Cl^- (123.8), HCO_3^- (21), HPO_4^{2-} (2.4), $H_2PO_4^-$ (0.6), glucose (10), mannose (10), glutamine (2.5), and β-hydroxybutyrate (0.5). The segments were permanently oxygenated with a mixture of 95% oxygen and 5% carbon dioxide (pH 7.4, 37 °C). After 30 min of equilibration, TEER was measured using bipolar rectangular current pulses (10 μA, 200 ms) and a programmable voltage-clamp device (Müssler Scientific Instruments).

In vitro myeloperoxidase activity measurement. The distal colon was cut into small pieces and homogenized in a 50 mM phosphate buffer, pH 6, with 0.5% cetrimonium bromide (50 mg tissue/ml of buffer) and incubated at 60°C for 2 hours. Myeloperoxidase (MPO) activity was assessed in a clear supernatant using 3,3',5,5'-tetramethylbenzidine (Sigma-Aldrich) as a substrate as described before[4]. Final activity is expressed in U per mg of protein.

Protein extraction and immunoblotting. Excised proximal and distal colons or ilea were cut open longitudinally and washed with PBS on ice. The colonic mucosa was scraped off using square coverslips. Snap-frozen mucosal scrapings were homogenized in ice-cold RIPA (20 mM Tris-HCl pH 7.5, 150 mM NaCl, 1 mM Na_2EDTA, 1 mM EGTA, 1% NP-40, 0.5% SDS supplemented with Halt protease and a phosphatase inhibitor Cocktail (Thermo Fisher Scientific)) using the Tissue Lyzer II

(Qiagen). Caco-2 cells were lysed in ice-cold RIPA by shearing through a 29G needle. Protein concentrations were determined using a BCA Protein Assay Kit (Thermo Fisher Scientific). Clarified lysates were resolved on SDS-PAGE and transferred to a nitrocellulose membrane for immunodetection. The following primary antibodies were used: ZO-1 (61-7300, ThermoFisher), E-cadherin (610181, BD Biosciences), desmoglein (611002, Progen), integrin α6 (ab181551, Abcam), integrin β4 (ab182120, Abcam), GAPDH (G9545, Sigma-Aldrich), keratin 8 (Troma I, Developmental Studies Hybridoma Bank), keratin 19 (Troma III, Developmental Studies Hybridoma Bank), and keratin 18 (Ks18.04, Progen). The following secondary antibodies were used: HRP-conjugated goat anti-guinea pig IgG (Sigma-Aldrich), donkey anti-mouse IgG (IRDye 680RD), donkey anti-rabbit IgG (IRDye 800CW), and goat anti-rat (IgG IRDye 800CW; all Licor). Signals were detected with an ECL Plus Western Blotting Detection System (GE Healthcare Life Sciences) and recorded with a Luminescent Image Analyzer LAS-3000 (Fujifilm Life Science, Düsseldorf, Germany) or the Odyssey 9120 imaging system (Licor). The densitometry of blots was analyzed using QuantiScan version 1.5 software (Biosoft).

Quantitative reverse transcriptase PCR. RNA was isolated from snap-frozen mucosal scrapings (see above) using TRI reagent (Sigma-Aldrich) according to the manufacturer's instructions. The RNA concentration was determined using the Nanodrop ND-1000 (Thermo Fisher Scientific). cDNA was prepared using M-MLV reverse transcriptase (ThermoFisher Scientific) with random oligo(dT)18 primers. qPCR was performed with SYBR Green JumpStart Taq ReadyMix (Sigma-Aldrich) using gene-specific primers (Supplementary Table 2). Due to previously reported instability of reference genes in colitic mice[5], expression of several reference genes was compared in $Ple^{fl/fl}$ and $Ple^{\Delta IEC}$ mice (Actb, Eef2, GAPDH, Hmbs and Tbp). As expression of none of these genes significantly differed between $Ple^{fl/fl}$ and $Ple^{\Delta IEC}$, relative RNA expression was calculated by the comparative threshold cycle method (ΔΔCt)[6] using a GAPDH internal reference gene control.

Cell stretching. Stretch experiments were carried out on flexible polydimethylsiloxane (PDMS, Sylgard) substrates with 4.0 cm^2 internal surface. The stretcher had a linear stage for a uniaxial stretch

and was driven by a computer-controlled stepper motor [7]. The substrates were coated with 50 μg/ml laminin or collagen type I in PBS at 4°C overnight, and 50,000 cells were seeded 24 hours prior to experiments. A uniaxial cyclic stretch was performed in an incubator under normal cell culture conditions (37°C, 5% CO_2, 95% humidity) for 1 hour at 10, 20, 30 and 50% stretch amplitude (peak-to-peak).

Radial shear assay. Radial shear assay was performed on a customized spinning disk device[8] consisting of a rotating glass plate driven by compressed air. The glass plate was located approximately 300 μm above a 35 mm plastic dish with adherent cells seeded at a density of 15000 cells/dish. The shear force was generated by a rotational speed of 1500 rpm and applied for 5 min. To assess the cell density, images of areas defined by radial distances 2-4 mm of the dish (corresponding to 0.7-1.5 Pa shear stress) were acquired before and after spinning. Then cells were stained with propidium iodide (PI; Fischer Scientific), and fractions of dead (PI-positive) and detached cells were calculated.

Magnetic tweezer microrheology. To determine the strength of cell-matrix adhesion, 5.09 μm carboxylated super-paramagnetic beads (microParticles GmbH) were coated with 20 μg/100 μl laminin or collagen type I (in PBS). The bead slurry (50%) was added to Caco2 and hCC cells grown on a 35 mm plastic dish and incubated for 1 hour under standard conditions. A magnetic field was generated as previously described[9] using a solenoid with a needle-shaped core (HyMu80 alloy, Carpenter). The needle tip was placed at a distance of 20 μm from a bead bound to the cell surface using a motorized micromanipulator (Injectman NI-2, Eppendorf). During measurements, bright-field images were taken by a CCD camera (ORCA ER, Hamamatsu) at a rate of 40 frames/s. The median of bead detachment (50% of adherent beads), determined under increasing forces of up to 15 nN, was used for calculating cumulative rupture forces.

Faecal microbiota analysis. Stool samples were collected from 6 $Ple^{fl/fl}$ and 6 $Ple^{\Delta IEC}$ mice at the age of 4, 12 and 20 weeks and analyzed for bacterial composition as described earlier[10]. Briefly, genomic DNA was extracted with a MasterPure™Complete DNA and RNA Purification Kit (Epicentre) with repeated

bead-beating in Lysing Matrix Y tubes using a FastPrep homogenizer (both MP Biomedicals). Next, the V3-V4 region of the 16S rRNA gene was amplified using barcoded bacterial 16SrRNA-specific primers 341F (5'-CCTACGGGNGGCWGCAG-3') and 806R (5'-GGACTACHVGGGTWTCTAAT-3'). PCR amplification was performed with KAPA 2G Robust Hot Start DNA Polymerase (Kapa Biosystems), with following concentrations: Buffer B 1×, Enhancer 1×, dNTP 0.2 mM each, primers 0.5 µM each, DNA sample 4 ng/µl, KAPA polymerase 0.5 U. Cycle parameters were 3 min 94°C, 25 cycles of 30 s at 94°C, 1 min at 54.2°C, and 1 min 15 s at 72°C; the final extension was at 72°C for 10 min. Three PCR products were pooled to minimize random PCR bias, and the length of PCR products was checked by agarose gel electrophoresis. Equal amounts of each sample were plate-purified using the SequalPrep™Normalization Plate (96) Kit (Invitrogen). Then equimolar amounts of PCR products from each sample were pooled, and MiSeq platform compatible adapters were ligated using a TruSeq DNA PCR-Free LT Kit (Illumina). The libraries were quantified using a KAPA Library Quantification Kit (Illumina) and sequenced on a MiSeq platform using a 2× 300bp kit at the CEITEC Genomics Core Facility.

Sequencing data were processed using QIIME (Quantitative Insights Into Microbial Ecology) version 1.9.1[11]. Quality filtering, chimera detection, read demultiplexing, and read clustering were done as described previously[12]. Raw reads were demultiplexed and quality filtered, and all sequences containing unknown base calls were excluded. Chimeric reads were detected and discarded using USEARCH algorithms[13]. The final dataset contained 80,185 high-quality reads (median 1,306 reads per sample, range 28 - 8766). To make samples comparable, 4 samples with fewer than 460 reads were removed from the analysis, and the OUT table was rarefied at a depth of 460 sequences per sample. Operational taxonomic units (OTUs) were clustered at a 97% similarity level, and representative sequences were identified using a Ribosomal Database Project classifier[14] against bacterial GreenGenes database 13.8[15]. For a microbiota analysis, PD whole tree metrics measuring the total descending branch length in the phylogenetic tree for each OTU was used to describe alpha diversity. The Principle Coordinate Analysis (PCoA) based on unweighted UniFrac distance metrics was used to describe beta diversity.

The sequence data are available in the Sequence Read Archive (SRA; http://www.ncbi.nlm.nih.gov/sra) under BioProject accession number PRJNA561691.

High salt extraction of Caco-2 cells. High salt extraction of Caco-2 cells was performed as described previously[16]. Cell fractions were prepared by solubilizing cells for 2 min at 4°C with a buffer containing 1% TX-100, 5 mM EDTA, and Halt Protease Inhibitor Cocktail (Thermo Fisher Scientific) in PBS pH 7.4, followed by centrifugation (16,000 × g, 10 min). The supernatant was collected as a soluble fraction. The pellet was homogenized in 1 ml of 10 mM Tris-HCl pH 7.6, 140 mM NaCl, 1.5 M KCl, 5 mM EDTA, 0.5% Triton X-100, supplemented with Halt Protease Inhibitor Cocktail. After 30 min (at 4°C), the homogenate was pelleted (16,000×g; 10 min), and the pellet (insoluble fraction) was rehomogenized with 5 mM EDTA in PBS pH 7.4. The resulting homogenate was further centrifuged (16,000×g; 10 min) to obtain the insoluble keratin-enriched high salt extract (HSE). All fractions were resolved by SDS-PAGE and their composition was analyzed by immunoblotting.

2. Supplementary TABLES

Supplementary Table 1

	N	Age* (years)	Gender	Duration * (years)	Extension	Treatment	Mayo† 0/1/2/3	p-ANCA† †	Inflammation† † † 0/1/2/3
healthy	20	46.75 (21-73)	11/9	-	-	-	-	-	-
UC	97	44.48 (19-87)	54/43	12.61 (1-37)	2/24/66	0/39/25/30	37/36/20/3	33	60/10/16/10

N: number of patients included in the analysis

*Mean (range)

Gender: Male/Female

Extension (UC): proctitis/left-sided/pancolitis

Treatment: None/Aminosalicylates (ASA)/Aminosalycilates + Azathioprine (ASA+AZA)/Biological treatment (Infliximab, Vedolizumab)

†Mayo referred to colonic segment used for the analysis

††p-ANCA: anti-neutrophil cytoplasm antibodies

†††Inflammation referred to colonic segment used for the analysis

Supplemental Table 1. *Clinical and demographic descriptions of patients with ulcerative colitis (UC) and healthy patients (healthy).*

Supplementary Table 2

	Forward (5'- 3')	Reverse (5'- 3')
plectin	CGCTGTGACAACTTCACCAC	CTCCAGGTTGGTCTGTCGAT
K18	TGAAGCGCTGGCTCAGAAGAAC	ACTGTGGTACTCTCCTCAATCTGC
K19	AGATCATGGCCGAGAAGAACCG	TGGGTGTTCAGCTCCTCAATCC
K8	AGGACTGACCGACGAGATCAAC	AACTCACGGATCTCCTCTTCATGG
GAPDH	AACTTTGGCATTGTGGAAGG	GTCTTCTGGGTGGCAGTGAT
Itgα3	ATCAACCAGGATGGATTCCAGGAC	TTTGCCCAAGCCCTCAAATGGG
Itgα5	ACTTGTCAGACACCCAGGGAAC	GCTCTGGTTCACAGCAAAGTAGTC
Itgα6	AAGACCAGTGGATGGGAGTCAC	TCATATCGATGTGCACACGTCACC
Itgβ1	TGTGGGTGGTGTACAAATACGAC	CATCACATCGTGCAGAAGTAGGC
Itgβ4	CCTCTGGATTCCTGTGCAATGACC	AACCAGGCTCACACACACACTC
Dsp1/2	TCAGAGCCATGACTATTGCCAAGC	TGGAGCTCAAGGTCTTCGATGG
Dsg2	ACCGCCTTTCGGCATATTCGTC	TCCAATGCATAGCCTGTCAGCAG
E-cad	TCATCGCCACAGATGATGGTTC	AACAGGACCAGGAGAAGAGTGC
ZO-1	TGCCCTGAAAGAAGCGATTCAGC	ACTTGTAGCACCATCCGCCTTC

Supplemental Table 2. *List of used RT-qPCR primers and their corresponding sequences.*

Supplementary Table 3

	Forward (5'- 3')	Reverse (5'- 3')
Atp5c1	GACCGCAAGGGATGGAAGAG	AGAATCCCCTTAGCAGCGTG
Gng2	GCCTGTGTCCTGTGGTTACA	TGCTGTAGAGACAGGGTGGT
Pwrn4	CCCCTATGACCAGAACGCAA	GCCAGCAGATGTTGTAACGG
Rab38	ACAAGTTGCTGGTGATTGGC	GCCTGGAACTATCCCGGTTT
Col20a1	TTCCGGAGATGGATTGGCG	TAAGTGGGCAGGATTGCGG
Sox13	TGTAGCCTTTCCGCCTGTTT	CCACTCTCCTCGGTGCAAAA
Supt6h	TCCCCCAGCATTTGACACAA	CACCGAAGATTTCCTGGGCT
Mmel1	GACATGGGGACGACAACACT	CCCCTCCGTTGTCAGCAAT

Supplemental Table 3. *List of potential off-target genes and PCR primers with their corresponding sequences.*

3. Supplementary REFERENCES

1. Brauer R, Tureckova J, Kanchev I, Khoylou M, Skarda J, Prochazka J *et al.* MMP-19 deficiency causes aggravation of colitis due to defects in innate immune cell function. *Mucosal immunology* 2015.

2. Chalupsky K, Kanchev I, Zbodakova O, Buryova H, Jirouskova M, Korinek V *et al.* ADAM10/17-Dependent Release of Soluble c-Met Correlates with Hepatocellular Damage. *Folia Biologica* 2013; **in press**.

3. Schindelin J, Arganda-Carreras I, Frise E, Kaynig V, Longair M, Pietzsch T *et al.* Fiji: an open-source platform for biological-image analysis. *Nat Methods* 2012; **9**(7)**:** 676-682.

4. Pulli B, Ali M, Forghani R, Schob S, Hsieh KL, Wojtkiewicz G *et al.* Measuring myeloperoxidase activity in biological samples. *PLoS One* 2013; **8**(7)**:** e67976.

5. Eissa N, Hussein H, Wang H, Rabbi MF, Bernstein CN, Ghia JE. Stability of Reference Genes for Messenger RNA Quantification by Real-Time PCR in Mouse Dextran Sodium Sulfate Experimental Colitis. *PLoS One* 2016; **11**(5)**:** e0156289.

6. Pfaffl MW. A new mathematical model for relative quantification in real-time RT-PCR. *Nucleic Acids Res* 2001; **29**(9)**:** e45.

7. Bonakdar N, Schilling A, Sporrer M, Lennert P, Mainka A, Winter L *et al.* Determining the mechanical properties of plectin in mouse myoblasts and keratinocytes. *Experimental cell research* 2015; **331**(2)**:** 331-337.

8. Branis J, Pataki C, Sporrer M, Gerum RC, Mainka A, Cermak V *et al.* The role of focal adhesion anchoring domains of CAS in mechanotransduction. *Sci Rep* 2017; **7:** 46233.

9. Kah D, Durrbeck C, Schneider W, Fabry B, Gerum RC. High-Force Magnetic Tweezers with Hysteresis-Free Force Feedback. *Biophysical journal* 2020; **119**(1)**:** 15-23.

10. Kostovcikova K, Coufal S, Galanova N, Fajstova A, Hudcovic T, Kostovcik M *et al.* Diet Rich in Animal Protein Promotes Pro-inflammatory Macrophage Response and Exacerbates Colitis in Mice. *Front Immunol* 2019; **10:** 919.

11. Caporaso JG, Kuczynski J, Stombaugh J, Bittinger K, Bushman FD, Costello EK *et al.* QIIME allows analysis of high-throughput community sequencing data. *Nat Methods* 2010; **7**(5)**:** 335-336.

12. Bajer L, Kverka M, Kostovcik M, Macinga P, Dvorak J, Stehlikova Z *et al.* Distinct gut microbiota profiles in patients with primary sclerosing cholangitis and ulcerative colitis. *World J Gastroenterol* 2017; **23**(25)**:** 4548-4558.

13. Edgar RC, Flyvbjerg H. Error filtering, pair assembly and error correction for next-generation sequencing reads. *Bioinformatics* 2015; **31**(21)**:** 3476-3482.

14. Wang Q, Garrity GM, Tiedje JM, Cole JR. Naive Bayesian classifier for rapid assignment of rRNA sequences into the new bacterial taxonomy. *Appl Environ Microbiol* 2007; **73**(16)**:** 5261-5267.

15. DeSantis TZ, Hugenholtz P, Larsen N, Rojas M, Brodie EL, Keller K *et al.* Greengenes, a chimera-checked 16S rRNA gene database and workbench compatible with ARB. *Appl Environ Microbiol* 2006; **72**(7)**:** 5069-5072.

16. Toivola DM, Zhou Q, English LS, Omary MB. Type II keratins are phosphorylated on a unique motif during stress and mitosis in tissues and cultured cells. *Molecular biology of the cell* 2002; **13**(6)**:** 1857-1870.

4. **Supplementary FIGURES**

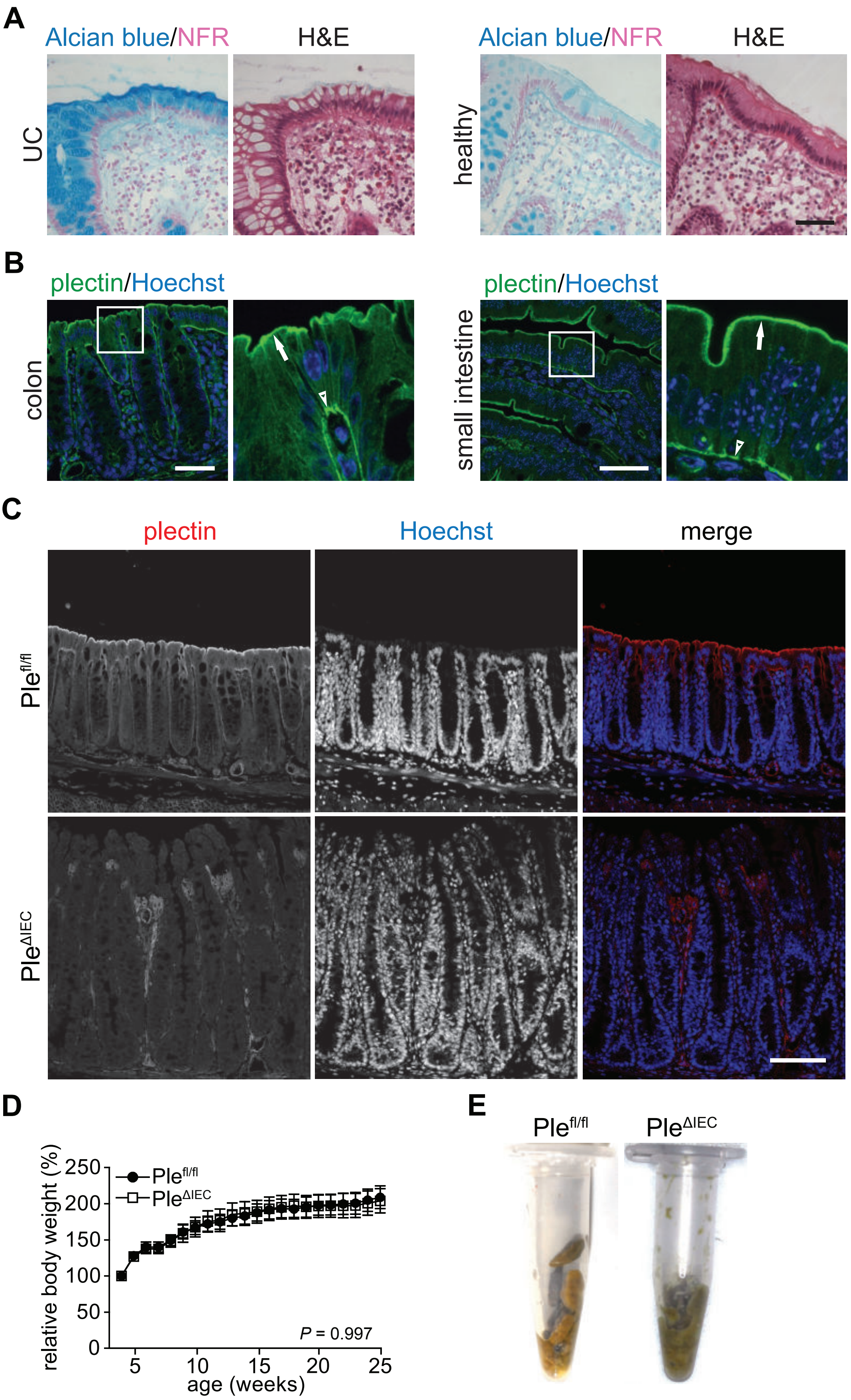

Figure S1

Figure S1 Immunolocalization of plectin in mouse intestine and phenotypic alterations of colonic epithelium, body weight, and stool consistency in plectin-deficient (*Ple*$^{\Delta IEC}$) mice. (A) Paraffin-embedded colon sections from UC patients (UC) and healthy controls (healthy) were stained with Alcian blue (mucus), nuclear fast red (nuclei), and H&E. Scale bar, 50 µm. (B) Paraffin-embedded sections from distal colon and small intestine of *Ple*$^{fl/fl}$ mice were immunolabeled with antibodies to plectin (green). Nuclei were stained with Hoechst (blue). Plectin staining at the apical (arrows) and basal (arrowheads) membranes. Scale bar, 50 µm. Boxed areas show ×4.5 images. (C) Paraffin-embedded distal colon sections from *Ple*$^{fl/fl}$ and *Ple*$^{\Delta IEC}$ mice were immunolabeled with antibodies to plectin (red). Nuclei were stained with Hoechst (blue). Scale bar, 100 µm. Note immunofluorescence signal in plectin-positive mesenchymal niche of both *Ple*$^{fl/fl}$ and *Ple*$^{\Delta IEC}$ colons. (D) Body weights of *Ple*$^{fl/fl}$ and *Ple*$^{\Delta IEC}$ mice were followed for 25 weeks. Graph shows relative body weight normalized to birth body weight. n = 7. (E) Stool consistency in *Ple*$^{fl/fl}$ and *Ple*$^{\Delta IEC}$ mice.

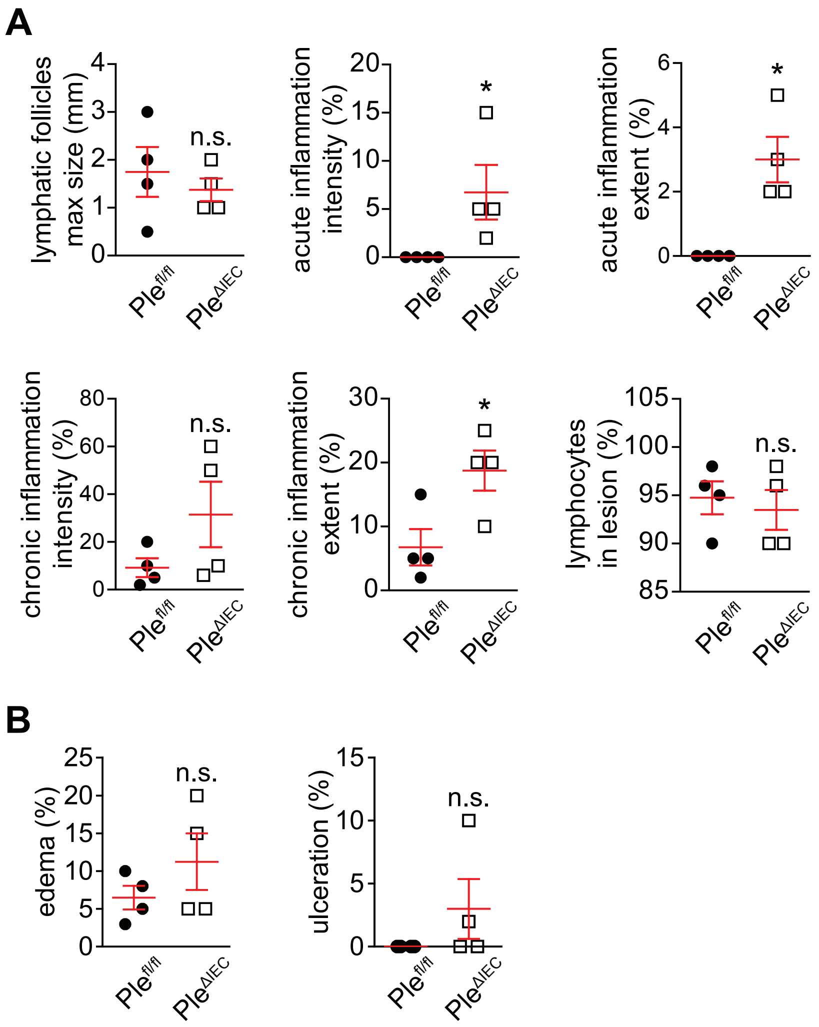
A
lymphatic follicles max size (mm)
n.s.
acute inflammation intensity (%)
*
acute inflammation extent (%)
*
chronic inflammation intensity (%)
n.s.
chronic inflammation extent (%)
*
lymphocytes in lesion (%)
n.s.
B
edema (%)
n.s.
ulceration (%)
n.s.
Ple fl/fl
Ple ΔIEC

Figure S2

Figure S2 Histological assessment of colonic tissue damage and inflammation in *Ple*$^{\Delta IEC}$ compared to *Ple*$^{fl/fl}$ mice. (A) Quantification of inflammatory parameters on H&E-stained sections of *Ple*$^{fl/fl}$ and *Ple*$^{\Delta IEC}$ colons. Graphs show maximal (max) sizes of lymphatic follicles, percentage of acute inflammation intensity, acute inflammation extent, chronic inflammation intensity, chronic inflammation extent, and lymphocytes in lesion. (B) Quantification of tissue damage assessed from H&E-stained sections of *Ple*$^{fl/fl}$ and *Ple*$^{\Delta IEC}$ colons (percentage of edema and ulceration). n = 4. Data are presented as mean ± SEM, n.s. not significant, *P < 0.05.

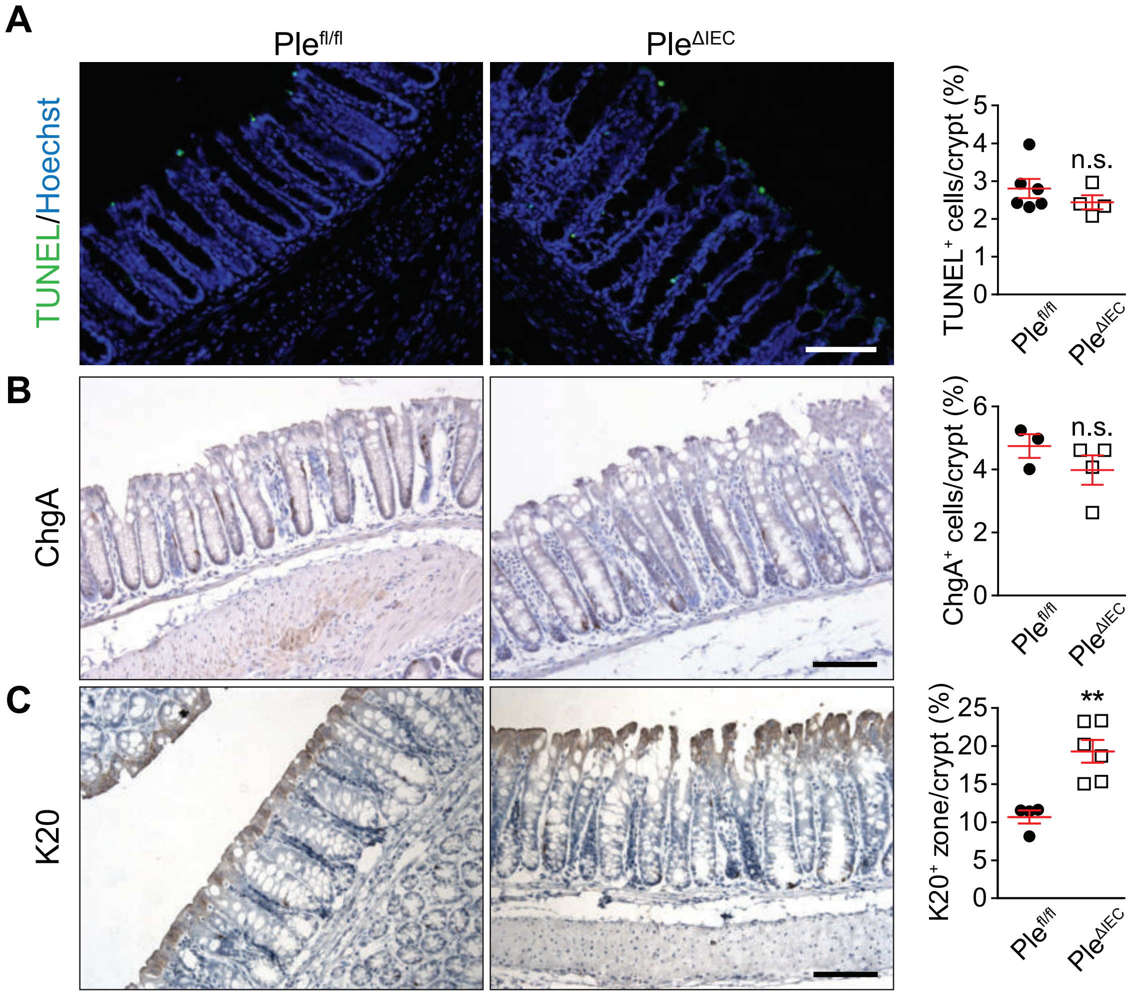
A
Ple$^{fl/fl}$
Ple$^{\Delta IEC}$
TUNEL/Hoechst
TUNEL$^+$ cells/crypt (%)
n.s.
B
ChgA
ChgA$^+$ cells/crypt (%)
n.s.
C
K20
K20$^+$ zone/crypt (%)
**

Figure S3

Figure S3 Characterization of colonic crypts in *Ple*$^{fl/fl}$ and *Ple*$^{\Delta IEC}$ mice. (A-C) Representative images of fluorescent TUNEL staining of apoptotic cells (green) (A), ChgA immunohistochemistry (enteroendocrine cells) (B), and K20 immunohistochemistry (mature IECs) (C) of *Ple*$^{fl/fl}$ and *Ple*$^{\Delta IEC}$ colon sections. Nuclei in (A) were stained with Hoechst (blue). Scale bars, 100 μm. Corresponding graphs show percentage of positively labeled (+) IECs and K20 zone per crypt cells. n = 3-6. Data are presented as mean ± SEM, n.s. not significant, $^{**}P < 0.01$.

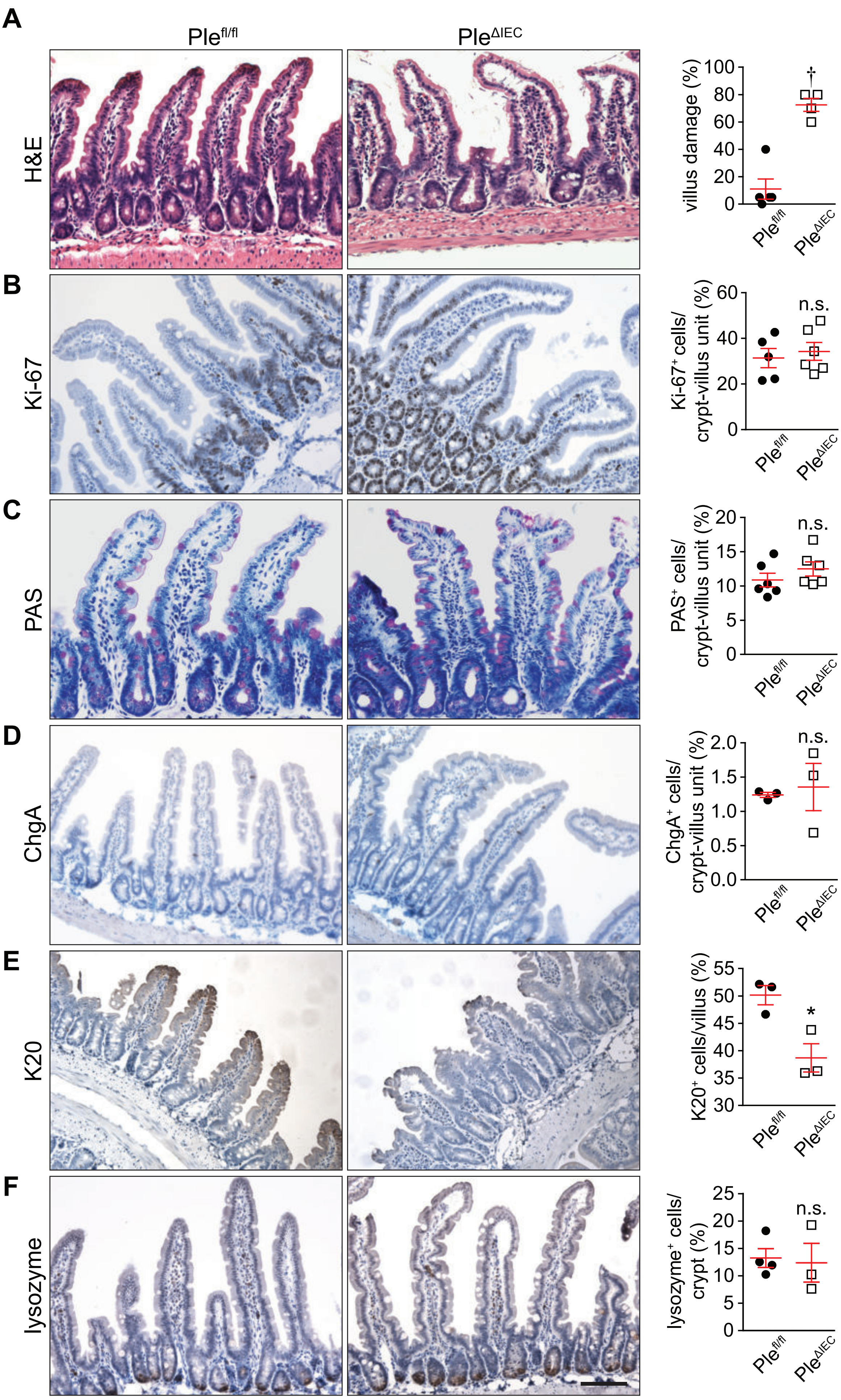
A
Ple fl/fl
Ple ΔIEC
H&E
villus damage (%)
100
80
60
40
20
0
†
Ple fl/fl
Ple ΔIEC
B
Ki-67
Ki-67+ cells/
crypt-villus unit (%)
60
40
20
0
n.s.
C
PAS
PAS+ cells/
crypt-villus unit (%)
20
15
10
5
0
n.s.
D
ChgA
ChgA+ cells/
crypt-villus unit (%)
2.0
1.5
1.0
0.5
0.0
n.s.
E
K20
K20+ cells/villus (%)
55
50
45
40
35
30
*
F
lysozyme
lysozyme+ cells/
crypt (%)
25
20
15
10
5
0
n.s.

Figure S4

Figure S4 Characterization of small intestinal crypt-villus units in *Ple*$^{fl/fl}$ and *Ple*$^{\Delta IEC}$ mice. (A-E) Representative images of H&E staining (A), Ki-67 immunohistochemistry (proliferating cells) (B), PAS staining (goblet cells) (C), ChgA immunohistochemistry (enteroendocrine cells) (D), K20 immunohistochemistry (mature IECs) (E), and lysozyme immunohistochemistry (Paneth cells) of small intestine sections. Scale bar, 100 µm. Corresponding graphs show percentage of damaged crypt-villus units (villus damage given as percentage of villi with > 10% of IECs detached from BM)A) and percentage of the positively labeled (+) IECs per crypt-villus unit (B, C), villus (D, E) and crypt (F). n = 3-6. Data are presented as mean ± SEM, n.s. not significant, *$P < 0.05$, †$P < 0.001$.

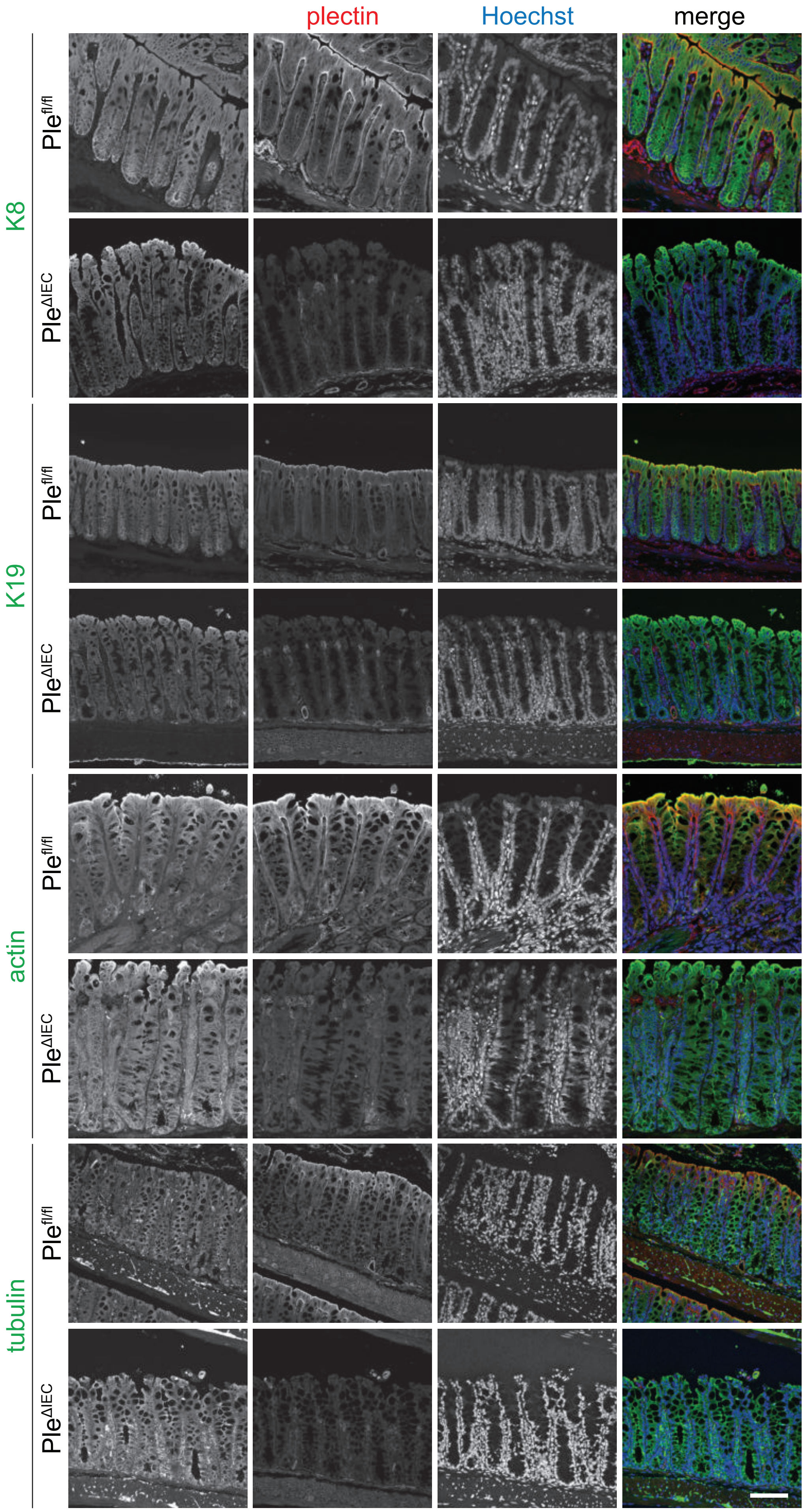
plectin
Hoechst
merge
K8
K19
actin
tubulin
Ple$^{fl/fl}$
Ple$^{\Delta IEC}$

Figure S5

Figure S5 Organization of KFs, actin fibers, and microtubules in colonic epithelium of *Ple*$^{fl/fl}$ and *Ple*$^{\Delta IEC}$ mice. Sections of *Ple*$^{fl/fl}$ and *Ple*$^{\Delta IEC}$ distal colon were immunolabeled with antibodies to K8, K19, β-actin, tubulin (all green), and plectin (red). Nuclei were stained with Hoechst (blue). Representative overview images are shown (see Figures 5A and S6A for more detailed images of pan-K- and β-actin-immunolabeled colons). Scale bar, 100 μm.

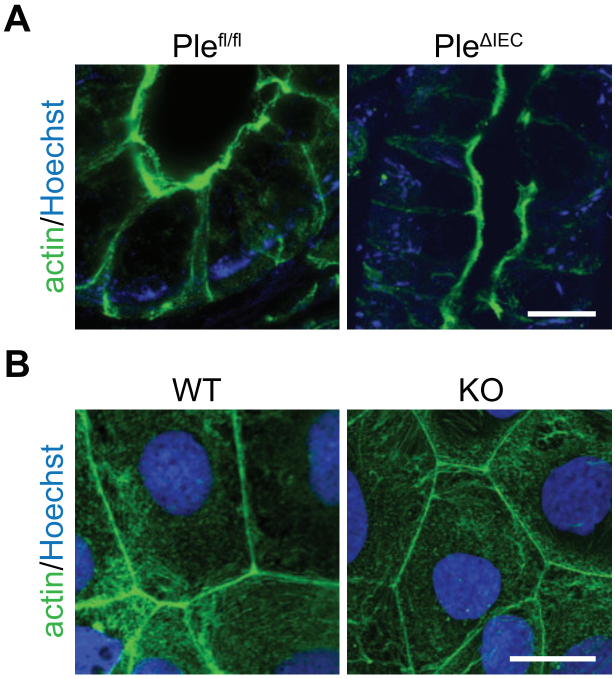

Figure S6

Figure S6 Comparable actin architecture in wild-type and plectin-deficient colonic epithelium and Caco-2 cells. (A) Representative super-resolution STED images of sections of *Ple*$^{fl/fl}$ and *Ple*$^{\Delta IEC}$ distal colon immunolabeled for β-actin. Nuclei were stained with Hoechst (blue). Scale bar, 10 μm. (B) Representative immunofluorescence images of WT and KO Caco-2 cell monolayer cultures immunolabeled for β-actin. Nuclei were stained with Hoechst (blue). Scale bar, 20 μm.

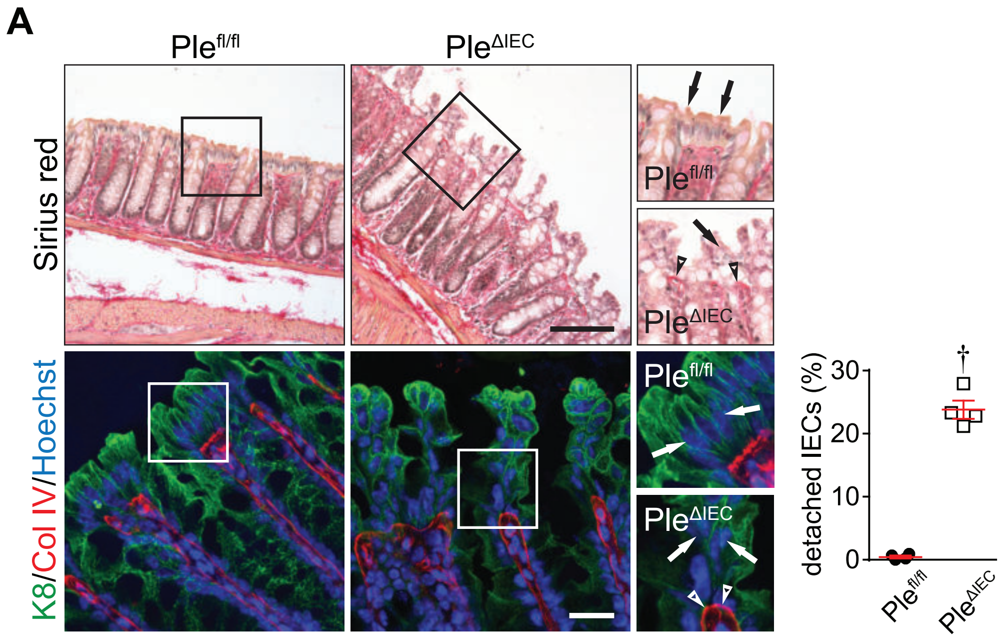

A
Ple^fl/fl
Ple^ΔIEC
Sirius red
K8/Col IV/Hoechst
Ple^fl/fl
Ple^ΔIEC
Ple^fl/fl
Ple^ΔIEC
detached IECs (%)
30
20
10
0
†
Ple^fl/fl
Ple^ΔIEC

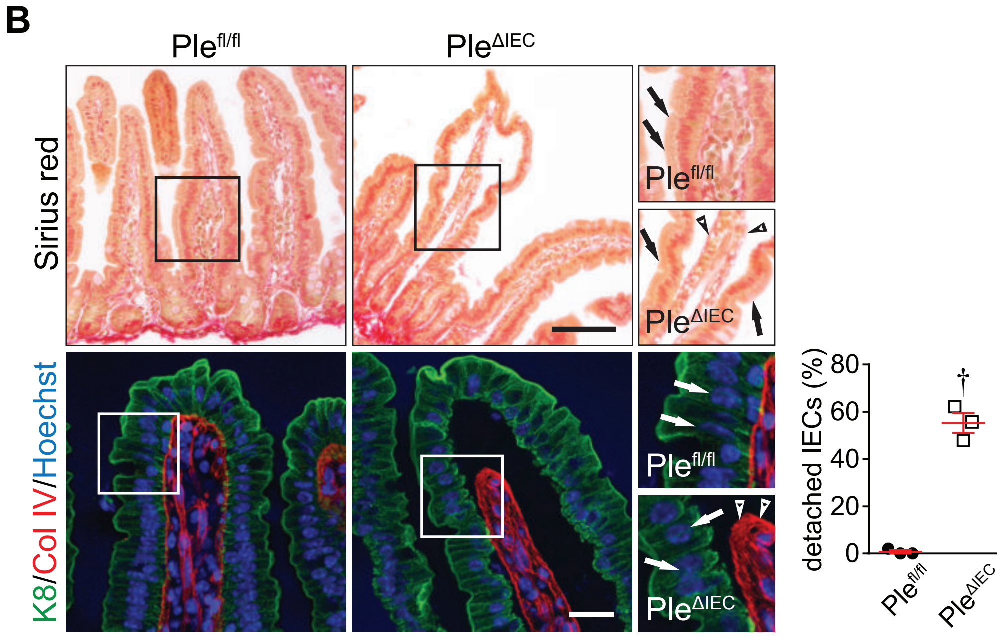

B
Ple^fl/fl
Ple^ΔIEC
Sirius red
K8/Col IV/Hoechst
Ple^fl/fl
Ple^ΔIEC
Ple^fl/fl
Ple^ΔIEC
detached IECs (%)
80
60
40
20
0
†
Ple^fl/fl
Ple^ΔIEC

Figure S7

Figure S7 Detachment of IECs from BM in *Ple*$^{\Delta IEC}$ intestinal epithelium. (A, B) Sections of *Ple*$^{fl/fl}$ and *Ple*$^{\Delta IEC}$ distal colon (A) and small intestine (B) were stained for fibrillar collagen with Sirius red (upper panels) and immunolabeled for K8 (green) and Col IV (red; lower panels). Nuclei were stained with Hoechst (blue). Boxed areas show ×1.5x images. The arrows, IECs; the arrowheads, BM. Scale bars, 100 μm (upper panels) and 25 μm (lower panels). Graphs show percentage of detached IECs per crypt (A) or villus (B). Data are presented as mean ± SEM, †$P < 0.001$.

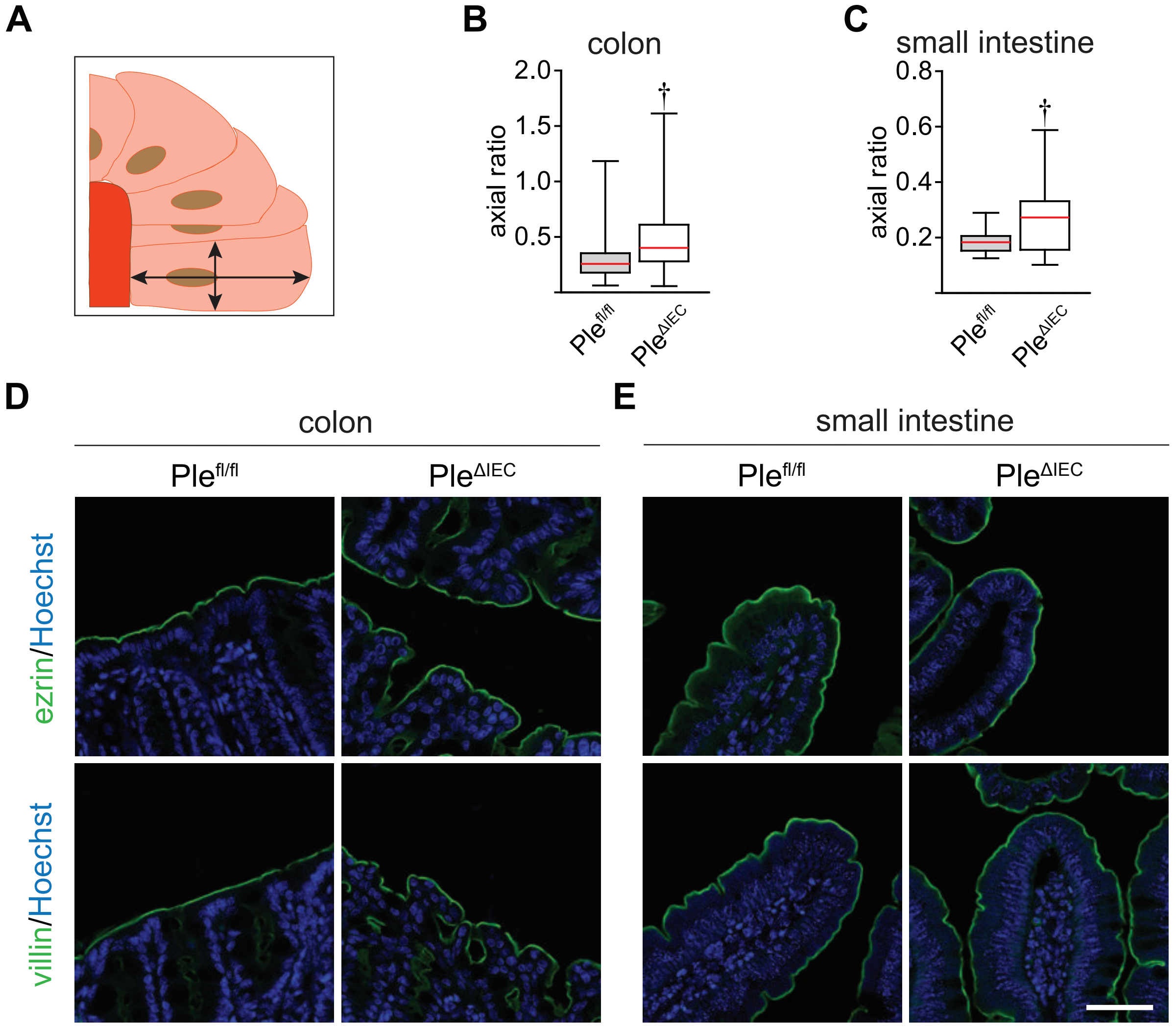
A
B
colon
axial ratio
2.0
1.5
1.0
0.5
Ple^fl/fl
Ple^ΔIEC
C
small intestine
axial ratio
0.8
0.6
0.4
0.2
Ple^fl/fl
Ple^ΔIEC
D
colon
Ple^fl/fl
Ple^ΔIEC
E
small intestine
Ple^fl/fl
Ple^ΔIEC
ezrin/Hoechst
villin/Hoechst

Figure S8

Figure S8 The $Ple^{\Delta IEC}$ IEC partially lose their apico-basal polarity, but retain a characteristic polarised distribution of the apical markers villin and ezrin. (A-C) Morphometric evaluation of $Ple^{fl/fl}$ and $Ple^{\Delta alb}$ IEC in K8- and Itgα6-immunolabeled colon (B) and small intestine (C) sections shown in Figure 5A. The axial ratio (the ratio between the largest and the smallest orthogonal cell diameter; indicated in A) was determined for >250 IEC in 3 mice per genotype. Boxplots show median, 25th, and 75th percentile with whiskers reaching to the last data point, †$P < 0.001$. (D, E) Representative immunofluorescence images of $Ple^{fl/fl}$ and $Ple^{\Delta IEC}$ distal colon (D) and small intestine (E) sections immunolabeled for ezrin (upper panels) and villin (lower panels; both green); Hoechst-stained nuclei (blue). Scale bar, 50 μm.

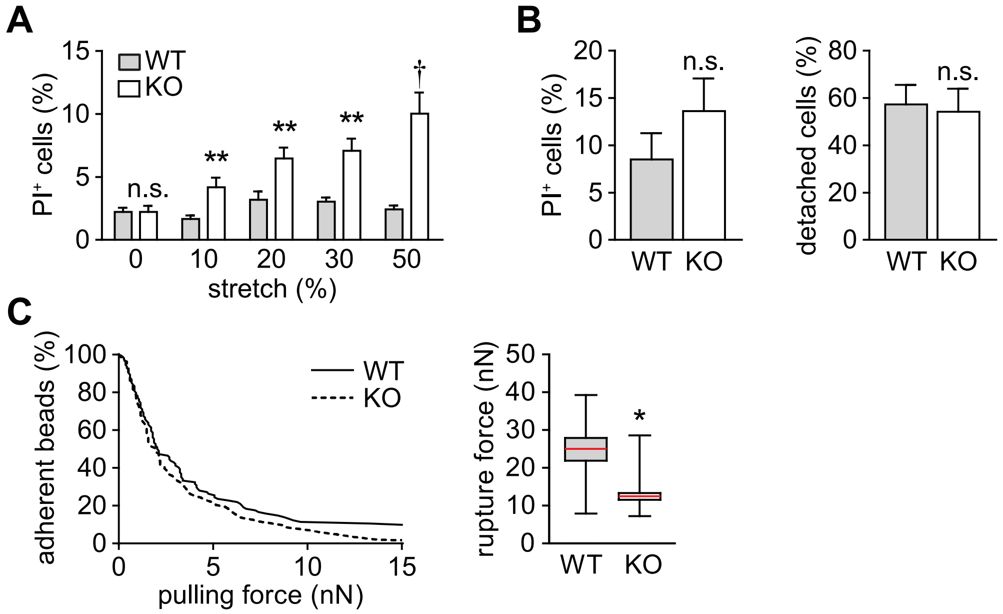
A
B
C
WT
KO
PI⁺ cells (%)
stretch (%)
n.s.
**
**
**
†
detached cells (%)
adherent beads (%)
pulling force (nN)
rupture force (nN)
*

Figure S9

Figure S9 Increased mechanical vulnerability and attenuated adhesion of KO hCC cells. (A) Quantification of viability of WT and KO hCC cells exposed to uniaxial cyclic stretch shown as percentage of dead (PI^+) cells. n = 9-12. (B) Quantification of WT and KO hCC cell viability (left) and adhesion (right) under radial shear flow shown as percentage of dead and detached cells, respectively. n = 8. (C) Adhesion strength between ECM-coated superparamagnetic beads and WT and KO hCC cells adhesions was quantified using magnetic tweezers that generated forces ramps at a speed of 1 nN/s up to a maximum force of 15 nN. The graph shows percentage of beads (n = 112 WT, 103 KO cells) that remained adherent at a given pulling force. Boxplot shows distribution of the 97.5 percentile detachment force (calculated from bootstrapping by sampling with replacement, n = 1000 runs) and its distribution (25th, and 75th percentile with whiskers reaching to the minimum and maximum sampled values). Bar graph data in all other subplots represent mean ± SEM, n.s. not significant, ***P* < 0.01, †*P* < 0.001.

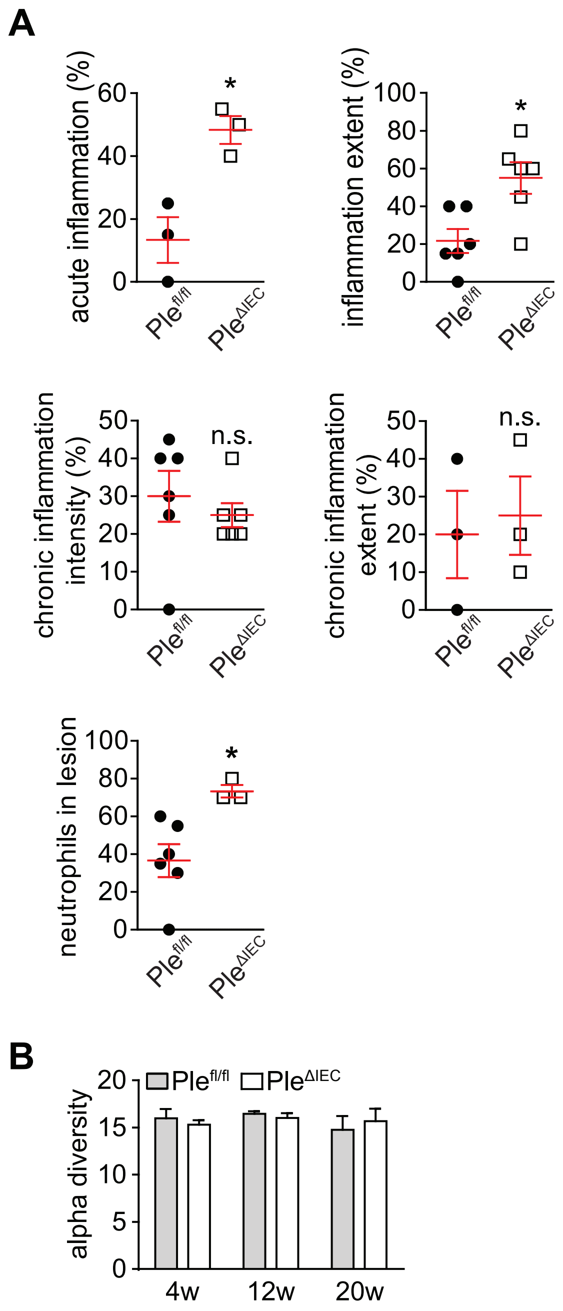
A
acute inflammation (%)
60
40
20
0
*
Ple fl/fl
Ple ΔIEC
inflammation extent (%)
100
80
60
40
20
0
*
chronic inflammation intensity (%)
50
40
30
20
10
0
n.s.
chronic inflammation extent (%)
n.s.
neutrophils in lesion
*
B
alpha diversity
20
15
10
5
0
4w
12w
20w

Figure S10

Figure S10 DSS-induced colitis in $Ple^{\Delta IEC}$ and $Ple^{fl/fl}$ mice. (A) Quantification of inflammatory parameters on H&E-stained colonic sections from DSS-treated $Ple^{fl/fl}$ and $Ple^{\Delta IEC}$ mice. Graphs show percentage of acute inflammation, inflammation extent, chronic inflammation intensity, chronic inflammation extent, and the number of neutrophils in lesion. n = 3-6.. (B) Alpha diversity of fecal microbiota in 4-, 12-, and 20-week-old untreated $Ple^{fl/fl}$ and $Ple^{\Delta IEC}$ mice determined by 16S rDNA sequencing using PD whole tree metrics. n = 4-6. Data are presented as mean ± SEM, n.s. not significant, $^*P < 0.05$.

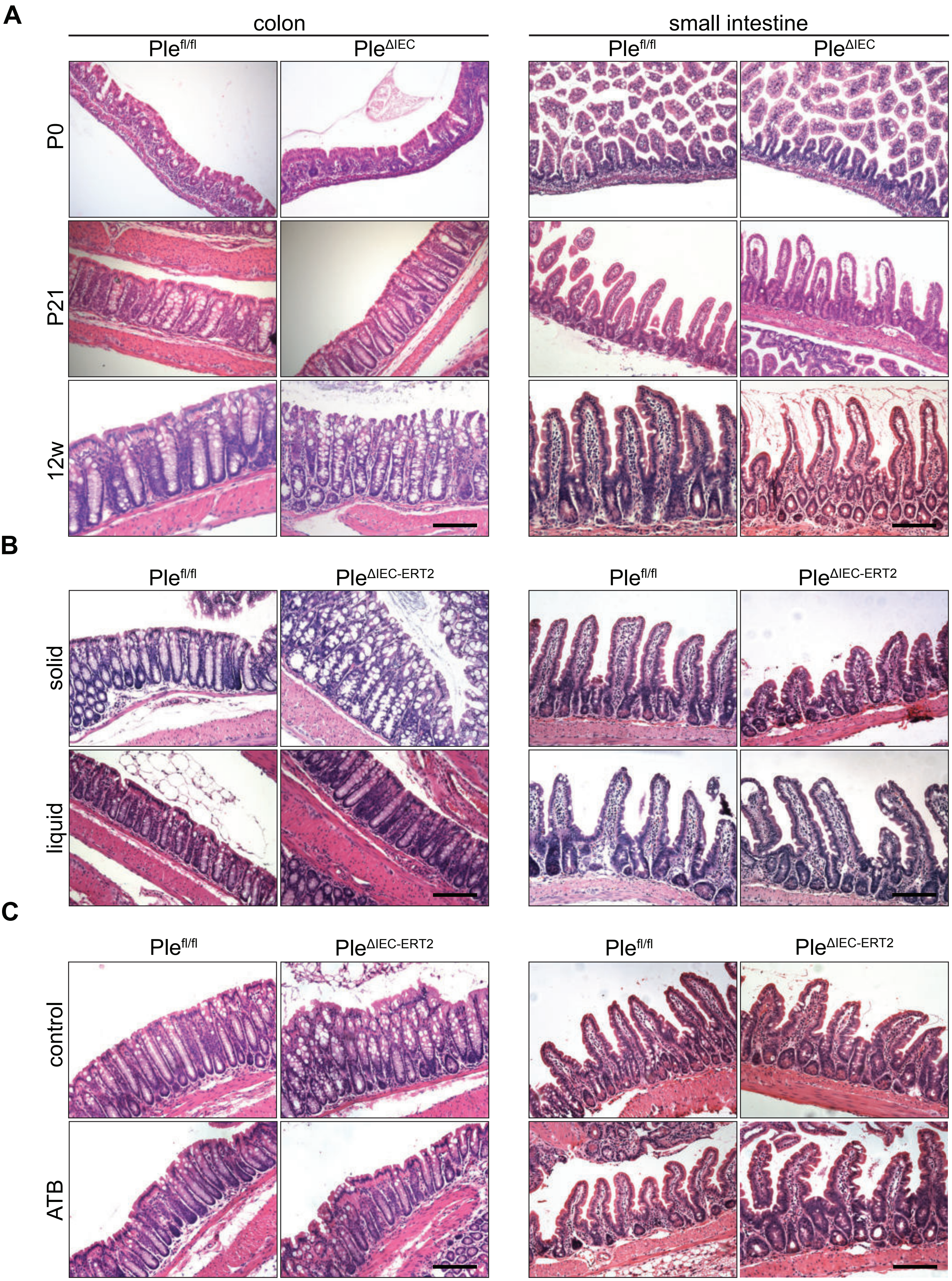

A
colon
small intestine
Ple fl/fl
Ple ΔIEC
P0
P21
12w
B
Ple fl/fl
Ple ΔIEC-ERT2
solid
liquid
C
Ple fl/fl
Ple ΔIEC-ERT2
control
ATB

Figure S11

Figure S11 Tissue damage in colon and small intestine of WT and plectin-deficient mice at weaning, on liquid diet, and under antibiotic treatment. (A) Representative images of H&E-stained colon (left panels) and small intestine (right panels) dissected from *Ple*$^{fl/fl}$ and *Ple*$^{\Delta IEC}$ mice at postnatal day 0 (P0), postnatal day 21 (P21), and at 12 weeks (12w). (B,C) Representative images of H&E-stained colon (left panels) and small intestine (right panels) dissected from 9-week-old *Ple*$^{fl/fl}$ and *Ple*$^{\Delta IEC\text{-}ERT2}$ mice, either kept on solid chow or provided with liquid diet (B) or exposed to antibiotic treatment (ATB) with broad-spectrum antibiotics (C) for 14 days. In all cases, plectin inactivation was induced by 3 consecutive applications of TMX on days 6, 8, and 10 and sacrificed on day 14. Scale bar, 100 μm.

A

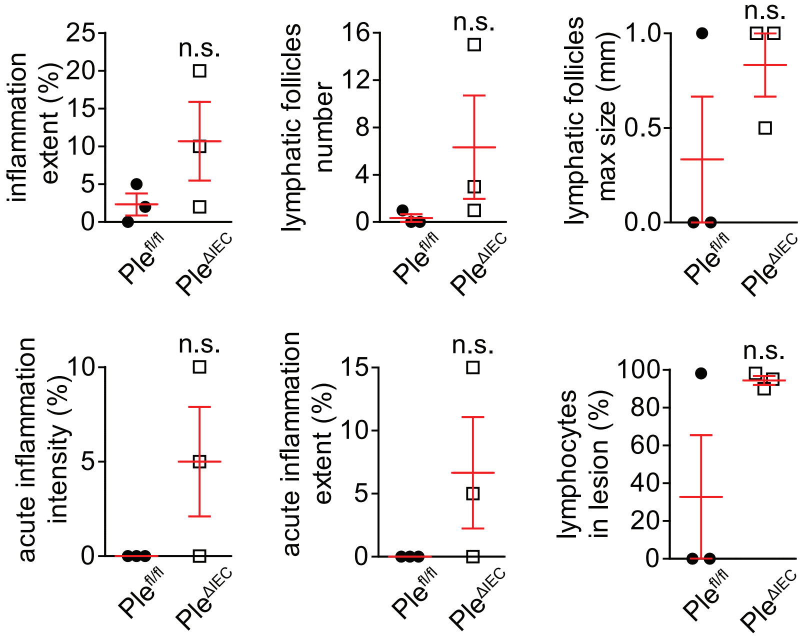

B

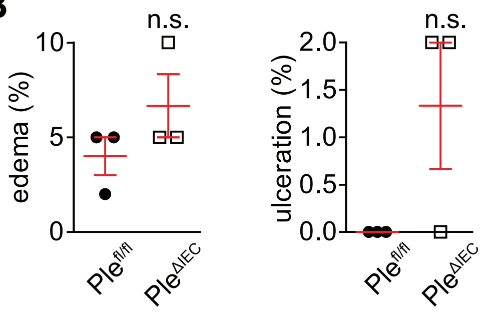

Figure S12

Figure S12 Histological assessment of colonic tissue damage and inflammation in 21-day-old *Ple*$^{\Delta IEC}$ compared to *Ple*$^{fl/fl}$ mice. (A) Quantification of inflammatory parameters on H&E-stained sections of *Ple*$^{fl/fl}$ and *Ple*$^{\Delta IEC}$ colons. Graphs show the number and maximal (max) sizes of lymphatic follicles, percentage of inflammatory extent, acute inflammation intensity, acute inflammation extent, and lymphocytes in the lesion. (B) Quantification of tissue damage assessed from H&E-stained sections of *Ple*$^{fl/fl}$ and *Ple*$^{\Delta IEC}$ colons (percentage of edema and ulceration). n = 3. Data are presented as mean ± SEM, n.s. not significant.

Caco-2

Intron 25-26

Atp5c1

WT	GCCTGCAAGGTGACGGGAGCCTGAGCAATAGCCCAGGGGAAGGTTAAGTCGT
KO	GCCTGCAAGGTGACGGGAGCCTGAGCAATAGCCCAGGGGAAGGTTAAGTCGT

WT

KO

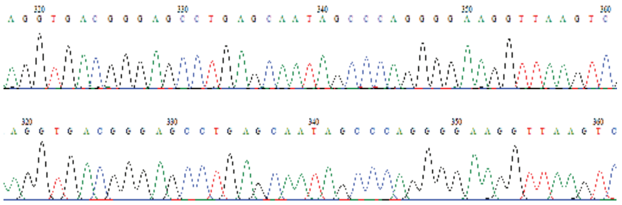

Gng2

WT	AAACTGTTAAATAACAGCTCCAGCAAAAGCCCAATGGAACAAACAAACAGCCAC
KO	AAACTGTTAAATAACAGCTCCAGCAAAAGCCCAATGGAACAAACAAACAGCCAC

WT

KO

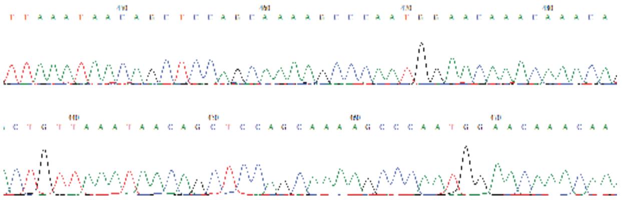

Pwrn4

WT	AACCACCAATCCCCATTGGGCTGCTGCTTGTGCCCACACTCGAAAAGCCAGAG
KO	AACCACCAATCCCCATTGGGCTGCTGCTTGTGCCCACACTCGAAAAGCCAGAG

WT

KO

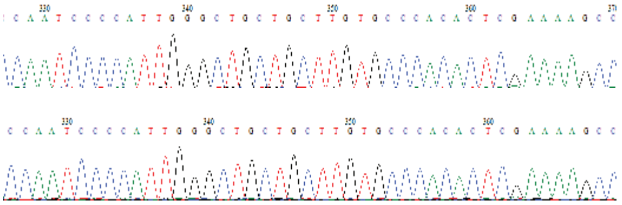

Rab38

WT	GTGCATTCCCTGGCGCTTTTGGGCTATTGCTTTAGCTCCTTCCCGCACAGTCCC
KO	GTGCATTCCCTGGCGCTTTTGGGCTATTGCTTTAGCTCCTTCCCGCACAGTCCC

WT

KO

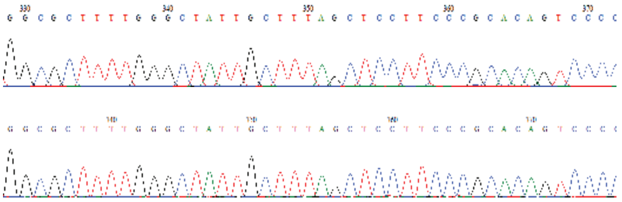

Exon 31

Col20a1

WT	AGCACCAGGTAACCTCTGCCTTCCTCAGGAAGCGCTGGGCAGGCGGACCCTGT
KO	AGCACCAGGTAACCTCTGCCTTCCTCAGGAAGCGCTGGGCAGGCGGACCCTGT

WT

KO

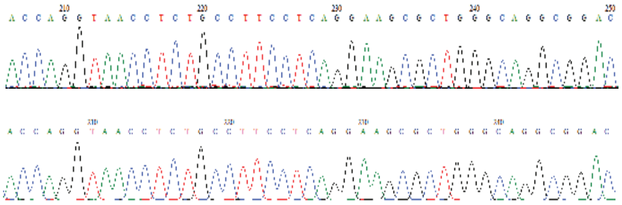

Sox13

WT	GGTCATCACACCCACCCACCTGCCTCCGGAAGAGCTGGGCTGGCTGGGGAGG
KO	GGTCATCACACCCACCCACCTGCCTCCGGAAGAGCTGGGCTGGCTGGGGAGG

WT

KO

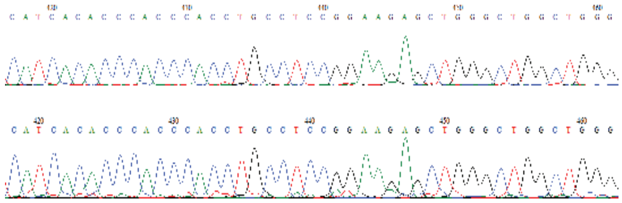

Supt6h

WT	TACCAGGCAGTGATTTCAGACTGCTCCCGGAAGCCCTGGGAGCTGTCTCTGCA
KO	TACCAGGCAGTGATTTCAGACTGCTCCCGGAAGCCCTGGGAGCTGTCTCTGCA

WT

KO

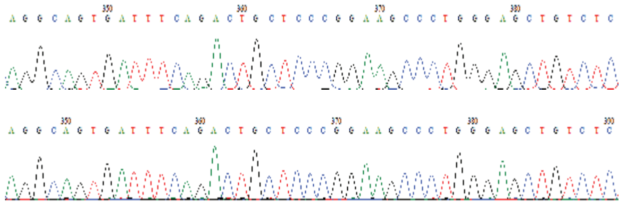

Mmel1

WT	AGTAACTTCTCCACCCAGCACTTCCGGGAGCAGTCAGAGTGCATGATCTACCAG
KO	AGTAACTTCTCCACCCAGCACTTCCGGGAGCAGTCAGAGTGCATGATCTACCAG

WT

KO

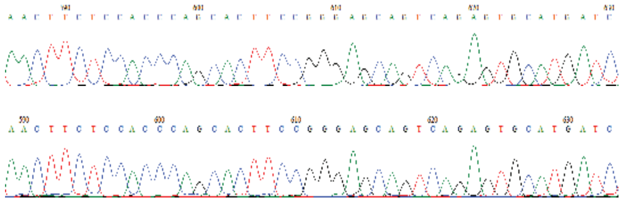

Figure S13

Figure S13 Analysis of potential off-target loci in Caco-2 cells. Four potential off-target loci for intron 25-26 (left panel) and exon 31 (right panel) guide RNA (gRNA) were amplified by PCR from genomic DNA of WT and plectin KO Caco-2 cells using gene-specific primers (listed in Supplemental Table 3). PCR products were analyzed by direct sequencing. Sequencing electropherograms and aligned sequences are shown. gRNA sequences in red; PAM sequences in blue.

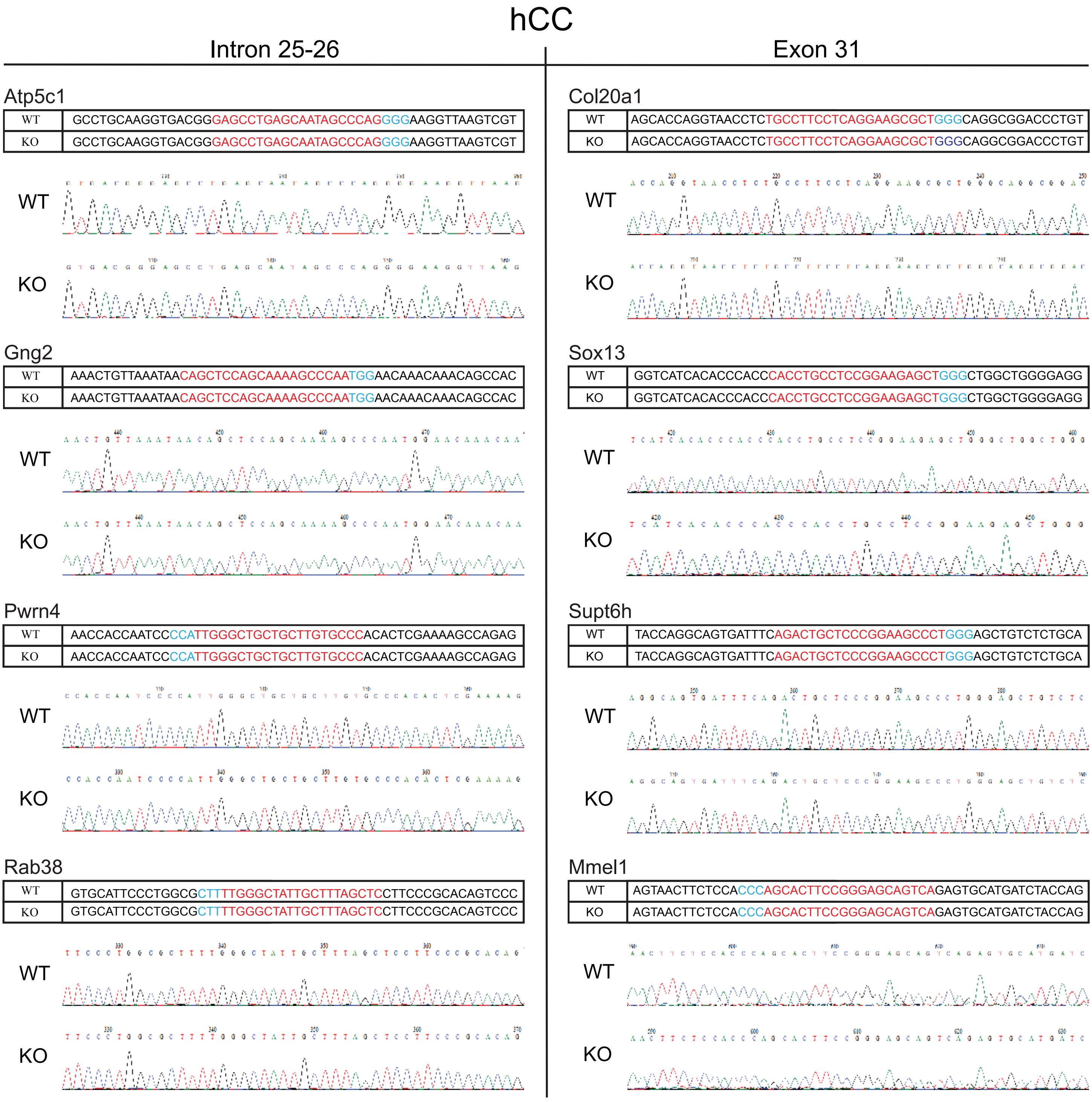
hCC
Intron 25-26
Exon 31
Atp5c1
WT GCCTGCAAGGTGACGGGAGCCTGAGCAATAGCCCAGGGGAAGGTTAAGTCGT
KO GCCTGCAAGGTGACGGGAGCCTGAGCAATAGCCCAGGGGAAGGTTAAGTCGT
WT
KO
Gng2
WT AAACTGTTAAATAACAGCTCCAGCAAAAGCCCAATGGAACAAACAAACAGCCAC
KO AAACTGTTAAATAACAGCTCCAGCAAAAGCCCAATGGAACAAACAAACAGCCAC
WT
KO
Pwrn4
WT AACCACCAATCCCCATTGGGCTGCTGCTTGTGCCCACACTCGAAAAGCCAGAG
KO AACCACCAATCCCCATTGGGCTGCTGCTTGTGCCCACACTCGAAAAGCCAGAG
WT
KO
Rab38
WT GTGCATTCCCTGGCGCTTTTGGGCTATTGCTTTAGCTCCTTCCCGCACAGTCCC
KO GTGCATTCCCTGGCGCTTTTGGGCTATTGCTTTAGCTCCTTCCCGCACAGTCCC
WT
KO
Col20a1
WT AGCACCAGGTAACCTCTGCCTTCCTCAGGAAGCGCTGGGCAGGCGGACCCTGT
KO AGCACCAGGTAACCTCTGCCTTCCTCAGGAAGCGCTGGGCAGGCGGACCCTGT
WT
KO
Sox13
WT GGTCATCACACCCACCCACCTGCCTCCGGAAGAGCTGGGCTGGCTGGGGAGG
KO GGTCATCACACCCACCCACCTGCCTCCGGAAGAGCTGGGCTGGCTGGGGAGG
WT
KO
Supt6h
WT TACCAGGCAGTGATTTCAGACTGCTCCCGGAAGCCCTGGGAGCTGTCTCTGCA
KO TACCAGGCAGTGATTTCAGACTGCTCCCGGAAGCCCTGGGAGCTGTCTCTGCA
WT
KO
Mmel1
WT AGTAACTTCTCCACCCAGCACTTCCGGGAGCAGTCAGAGTGCATGATCTACCAG
KO AGTAACTTCTCCACCCAGCACTTCCGGGAGCAGTCAGAGTGCATGATCTACCAG
WT
KO

Figure S14

Figure S14 Analysis of potential off-target loci in hCC cells. Four potential off-target loci for intron 25-26 (left panel) and exon 31 (right panel) guide RNA (gRNA) were amplified by PCR from genomic DNA of WT and plectin KO hCC cells using gene-specific primers (listed in Supplemental Table 3). PCR products were analyzed by direct sequencing. Sequencing electropherograms and aligned sequences are shown. gRNA sequences in red; PAM sequences in blue.
